# A ‘reader’ unit of the chemical computer

**DOI:** 10.1098/rsos.171495

**Published:** 2018-01-10

**Authors:** Pavel S. Smelov, Vladimir K. Vanag

**Affiliations:** Centre for Nonlinear Chemistry, Immanuel Kant Baltic Federal University, 14 A. Nevskogo Street, Kaliningrad 236041, Russia

**Keywords:** coupled oscillators, chemical computer, mode recognition

## Abstract

We suggest the main principals and functional units of the parallel chemical computer, namely, (i) a generator (which is a network of coupled oscillators) of oscillatory dynamic modes, (ii) a unit which is able to recognize these modes (a ‘reader’) and (iii) a decision-making unit, which analyses the current mode, compares it with the external signal and sends a command to the mode generator to switch it to the other dynamical regime. Three main methods of the functioning of the reader unit are suggested and tested computationally: (a) the polychronization method, which explores the differences between the phases of the generator oscillators; (b) the amplitude method which detects clusters of the generator and (c) the resonance method which is based on the resonances between the frequencies of the generator modes and the internal frequencies of the damped oscillations of the reader cells. Pro and contra of these methods have been analysed.

## 1. Introduction

An enormous number of works have been devoted to the study of the principles of the brain functioning. Recently, a new direction has been crystallized out, which is born at the intersection of the theory of dynamical systems and neural networks [1]. Coupling of the brain dynamics and brain connectivity (connectome) leads to the so-called ‘dynome' which can shed light on the brain architecture, functional organization of neuro-circuits and roles of different connections in a neural network [2].

It was demonstrated theoretically that the initially homogeneous medium of oscillators can be self-structured to perform certain functions [3,4]. This self-organization occurs, for example, due to changes in synaptic weights (plasticity). Buzsaki [3] offers general theories for the brain functioning. He focuses on the fact that there should be a ‘reading' system in the brain, a system which reads information from a special part of neural network. The latter can be conventionally called ‘central pattern generator' (CPG) [5], but this CPG is not necessarily linked to locomotion. Buzsaki believes that the ‘reader' sends a signal further to an integrator that collects information about the external objects. In general, several CPGs and several ‘readers' can coexist and work together.

Such a view on the architecture of the brain is similar to our approach to developing a ‘chemical computer', a system of coupled chemical oscillators that can perform such functions as signal (=image) recognition and decision-making or an adaptive and smart response to external stimuli. As a chemical oscillator, we use the Belousov–Zhabotinsky (BZ) reaction [6,7] in our laboratory experiments. The BZ reaction is a catalysed oxidation of malonic acid by bromate in an acidic environment. As a cell (or reactor) for the BZ reaction, we explore usual macro-reactors (continuously stirred tank reactors (CSTR) [8]) or micro-reactors which are water microdroplets (around 100 µm in diameter) in the oil phase [9]. The dynamics of the BZ reaction is similar to the dynamics of spiking neurons. We explore the BZ reaction both in the oscillatory mode and at the excitable stationary states. Sometimes we use a term ‘chemical neuron' for microdroplets with the BZ reaction inside.

To construct a network of the BZ cells similar (in some sense) to the neuronal network, we invented pulse coupling between the BZ cells with time delay [10,11] instead of habitual diffusive coupling. A few almost identical BZ oscillators with either inhibitory or excitatory pulsatile coupling with time delay can produce a lot of different dynamical modes [12,13]. Like in biology, we call this network of oscillators the CPG. Following Buzsaki's idea about a ‘reader', there should be some analysing block (a group of other BZ elements) that can distinguish between different modes of the CPG and send a corresponding signal to the other, let us say, logical or decision-making block of our chemical computer.

**Figure 1.**
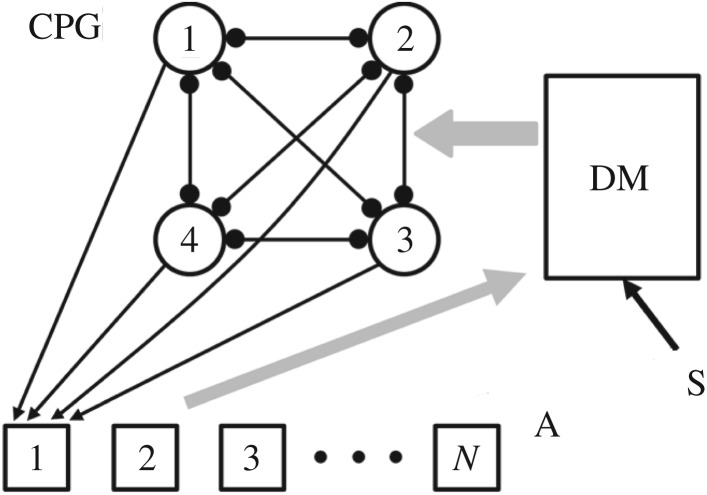
The hierarchical network of coupled oscillators which represents basic blocks of the ‘chemical computer’. Pulse-coupled spike oscillators 1–4 (circles) constitute the central pattern generator (CPG). Excitable cells of block A (from 1 to *N*, in squares) are elements that analyse dynamical modes of the CPG. The DM is the decision-making unit. External signal S is analysed by the DM unit. The CPG oscillators are connected to all A cells by unidirectional excitatory pulses. Links between the CPG oscillators and only the first A cell are drawn, to make the scheme readable.

Schematically, a block-scheme of the chemical computer is presented in figure 1. Pulse-coupled oscillators 1, 2, 3 and 4 (in circles) generate different modes of the CPG. When we take into account permutations of the CPG oscillators, then each mode (except one completely symmetrical mode) splits into sub-modes. Each CPG oscillator sends excitatory pulses to each of the *N* excitable elements of the analysing block A. Block (or unit) A can work in different ways, for example, only one excitable cell or a set of special cells from block A should be excited by the corresponding sub-mode for which they are tuned. All other elements of block A should remain in the steady state (i.e. be inactive). Block A, in turn, sends information about the state of the CPG to the next ‘decision-making' (DM) block (or unit). External signal S is also recorded by the DM block. Comparing signal S and the signal from block A, the DM unit should make a decision what to do with the current mode of the CPG. One of the possible results of this decision can be a switch from the current CPG mode to the other one. The DM block should consist, in general, of coupled oscillators or excitable cells as well. A feedback from the CPG via the ‘reader' and the DM units back to the CPG should create conditions for adaptive and smart behavior of the entire ‘chemical computer'. Logical functions can be employed in this feedback. Note that logic gates were created recently with the aid of the BZ droplets [14,15].

The ‘chemical computer' described above and the principals of its functioning are completely different from the features of the Hopfield networks [16] and from the principals of ‘computing with chemical waves' [14,17,18]. In our case, the dynamic modes of the CPG and switching between them are the essential part of an entire computer. Switching between the CPG modes is akin to heteroclinic computing [19,20]. We are working with relatively small circuits and believe that the principals of their functioning are quite instructive and can be applied to larger networks [21].

In the present work, we investigate computationally different methods of functioning the analysing block A that should differentiate between different modes of the CPG. Any oscillatory pattern (including CPG modes) can be characterized by the phases, amplitudes and frequencies of oscillations. Correspondingly we develop three methods that are based (a) on the phase differences between the CPG oscillators, (b) on the aggregated amplitudes of the oscillators in clusters, and (c) on resonances.

**Figure 2.**
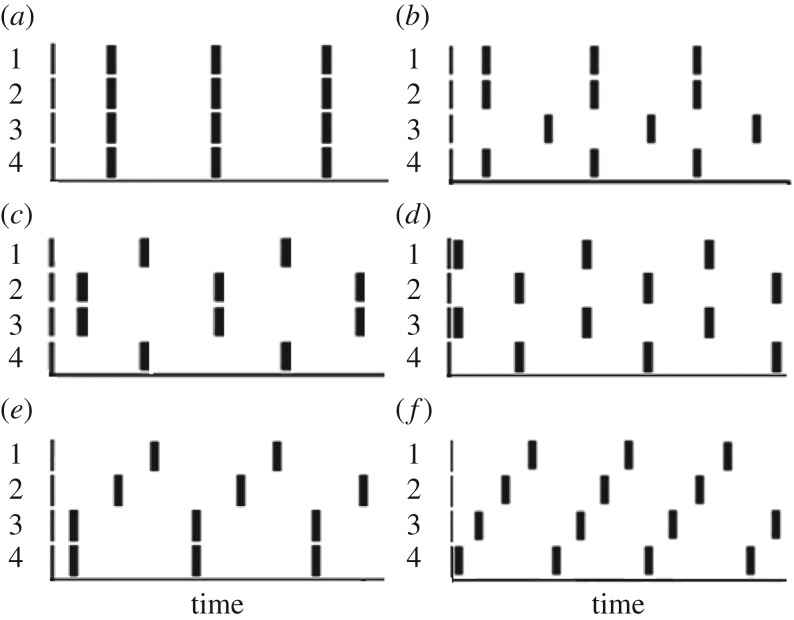
Regular modes in a network of four pulse-coupled oscillators: (*a*) IP, (*b*) ‘3 + 1’, (*c*) IPAP or AP(2,3 + 1,4), (*d*) AP, (*e*) ‘1 + 1+2’, (*f*) splay (walk). The time intervals of all panels are 400 s.

We use four oscillators with pulsatile all-to-all coupling as a CPG and analyse different (five) regular (R) modes found in this system (see figure 2) [12,13]. The first of these five R-modes is the symmetrical in-phase (IP) mode shown in figure 2*a*, when all four oscillators are in-phase. There are no sub-modes for the IP mode. In figure 2*b*, we present the so-called ‘3 + 1' mode (or triplet–singlet), when one cluster consists of three in-phase oscillators and the fourth oscillator, which is almost anti-phase (AP) to those three. There are four sub-modes of the ‘3 + 1' mode. The AP mode (which is another two-cluster mode), when two clusters of two IP oscillators oscillate AP, is exhibited by two sub-modes in figure 2*c*,*d*. There are three AP sub-modes obtained by permutation. These sub-modes have identical dynamics, but the reader unit should be able to recognize them. The ‘2 + 1 + 1' mode (three-cluster mode or doublet–singlet–singlet), where two oscillators are in-phase, while the phases of other two oscillators are shifted in time approximately by *T*/3 and 2 *T*/3 (or −*T*/3), where *T* is the global period of this mode, is shown in figure 2*e*. Experimentally this mode was found only recently [22,23]. In [22], this mode is called the minimum chimera. Finally, a splay (S) mode, when the phases of all oscillators are shifted in time by *T*/4, is shown in figure 2*f*. For the S mode, each oscillator can be considered as a ‘cluster' of one (singlet). If permutations are taken into account, there are twelve ‘2 + 1 + 1' modes and six S modes. There are 26 modes in total.

R-modes are found in such quadruped gates as walk (four-beat gait, when four legs move alternatively with a quarter period phase shift between legs), pace (a lateral, two-beat gait) or bound (when front legs move in-phase and back legs move in-phase and front and back pairs of legs move AP), trot (a diagonal, two-beat gait) and pronk (when all four legs move in-phase) [24–26]. Block A should inform the DM unit about each of these modes without mistakes.

The rest of this paper is organized as follows. In §2, we describe our equations and methods. In §3, we describe three different methods for identification of the different dynamical modes. In §4, we discuss our results.

## 2. Material and methods

To simulate the BZ reaction in all oscillatory cells of the CPG and in the excitable cells of the analysing block A (A cells), we explore our previous four-variable model [27]:

2.1 $$\begin{array}{r} \frac{dx}{dt}=-k_{1}xy+k_{2}y-2k_{3}x^{2}+k_{4}x(c_{0}-z)/(c_{0}-z+c_{\min})-k_{0}x\equiv G(x,y,z), \end{array}$$

2.2 $$\begin{array}{r} \frac{dy}{dt}=-k_{1}xy-k_{2}y+k_{9p}vz-k_{0}(y-y_{0})\equiv F(x,y,z,v), \end{array}$$

2.3 $$\begin{array}{r} \frac{dz}{dt}=2k_{4}x(c_{0}-z)/(c_{0}-z+c_{\min})-k_{9p}vz-k_{10}z\equiv P(x,z,v) \end{array}$$

2.4 $$\begin{array}{r} \;\mathrm{and}\;\frac{dv}{dt}=2k_{1}xy+k_{2}y+k_{3}x^{2}-k_{9p}vz-k_{13}v-k_{0}v\equiv W(x,y,z,v), \end{array}$$

where *x* is the concentration of activator, [HBrO_2_], *y* = [Br^−^] (inhibitor), *z* = [oxidized state of the catalyst], *v* = [BrMA] (bromomalonic acid), *c*_0_ is the total concentration of the catalyst (oxidized + reduced states), $c_{\min}\ll c_{0}$; $k_{1}=k_{1}^{\prime }h$, $k_{2}=k_{2}^{\prime }h^{2}A$, and $k_{4}=k_{4}^{\prime }hA$*, A* ≡ [NaBrO_3_] = 0.25 M, *h* = [H^+^], [MA] = 0.1 M, *c*_0_ = 1 mM, *k*_0_ = 5 × 10^−4^ s^−1^, $k_{1}^{\prime }=2\times 10^{6}\;M^{-2}\;s^{-1}$, $k_{2}^{\prime }=2\;M^{-3}\;s^{-1}$, *k*_3_ = 3000 M^−1 ^s^−1^, $k_{4}^{\prime }=42\;M^{-2}\;s^{-1}$, *k*_9p_ = 20 M^−1 ^s^−1^, $k_{10}=k_{10}^{\prime }[\mathrm{MA}]$, $k_{10}^{\prime }=0.05\;M^{-1}\;s^{-1}$, *k*_13_ = 0.004 s^−1^, *c*_min_ = (3*k*_r_*k*_10_*c*_0_)^1/2^/*k*_red_, *k*_r_ = 2 × 10^8^ M^−1 ^s^−1^, *k*_red_ = 5 × 10^6^ M^−1 ^s^−1^. Parameters *h* and *y*_0_ are used to tune the state of the BZ oscillator.

For the CPG oscillators, we use inhibitory pulse coupling with time delay. In this case, equation (2.2) in model (2.1)–(2.4) is modified as follows:

2.5 $$\frac{dy_{i}}{dt}=F(x_{i},y_{i},z_{i},v_{i})+\sum_{j\neq i}\left[C_{\mathrm{inh}}\times P(x_{j},\tau ,\Delta t)\right],$$

where *i* = 1, 2, 3, 4 and *j* = 1, 2, 3, 4; *C*_inh_ is the coupling strength, which can be interpreted as the rate (M/s) of injection of inhibitor. Rectangular function *P*(*x_j_*,*τ*,Δ*t*) switches *τ* seconds after a sharp spike of *x_j_* from 0 to 1 and then switches back to 0 after a time Δ*t* (=5 s in our case). Function *P*(*x_j_*,*τ*,Δ*t*) simulates, for example, a pulse injection of a solution of inhibitor (Br^−^) into the reactor or a flash of light that produces inhibitor inside a reactor. During one pulse, the concentration of inhibitor in the cell increases by *C*_inh_Δ*t*. All CPG oscillators are identical, *h* = 0.3 M and *y*_0_ = 0. In §3.3, we use *h* = 0.29 M to fulfil the resonance conditions better.

Between the CPG oscillators and A cells we use unidirectional excitatory pulse coupling with time delay. In this case, modification of equation (2.2) in model (2.1)–(2.4) and introduction of the fifth variable [Ag*_m_*] (silver ions) with the corresponding differential equation look as follows [27]:

2.6 $$\frac{dy_{m}}{dt}=F(x_{m},y_{m},z_{m},v_{m})-k_{\mathrm{diff}}[Ag_{m}]y_{m}$$

and

2.7 $$\frac{d[Ag_{m}]}{dt}=\sum_{i}\left[C_{\mathrm{ex}}\times P(x_{i},\tau_{im},\Delta t)\right]-k_{\mathrm{diff}}[Ag_{m}]y_{m},$$

where *m* = 1, 2, 3, … , *N* (numbering of A cells); *i* = 1, 2, 3, 4 (numbering of the CPG oscillators), *τ_im_* is the time delay between a spike in the *i*th oscillator and the pulsed perturbation of the *m*th A cell. Silver ions react with bromide ions very quickly (with the diffusion-controlled rate constant *k*_diff_ = 10^8^ M^−1 ^s^−1^), thus reducing *y_m_* in the *m*th cell. All A cells are in the excitable steady state, which is established by non-zero parameter *y*_0_.

**Figure 3.**
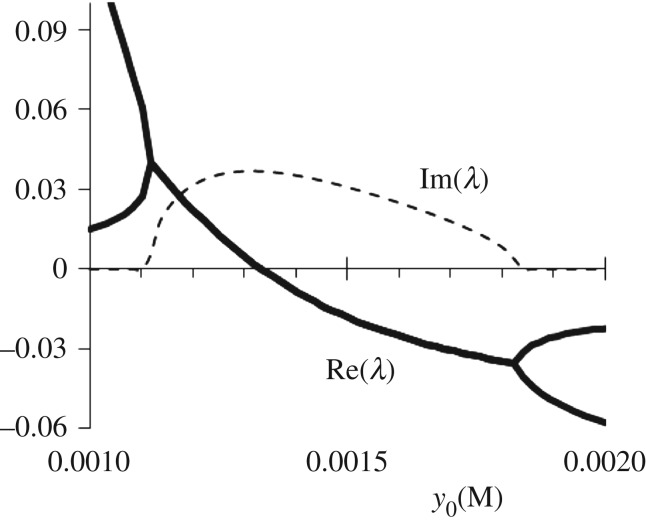
A typical dependence of the largest eigenvalues *λ* on *y*_0_ for the linearized system (2.1)–(2.4). Bold curves are Re(*λ*), while dashed curve is Im(*λ*). *h* = 0.3 M. All other parameters of system (2.1)–(2.4) are in the text.

The value of *y*_0_ should be as large as is needed to suppress oscillations (i.e. to move the system below the Hopf bifurcation). The parameters of the Hopf bifurcation are found by linear stability analysis of system (2.1)–(2.4) (see figure 3). At chosen parameters of the system, the value of *y*_0_ corresponding to the Hopf bifurcation is equal to ${y_{0}}^{H}$ (≅1.3331 mМ). At $y_{0}>{y_{0}}^{H}$, the oscillations are suppressed. The difference between the steady-state (SS) value *y*_SS_ (which is a function of *y*_0_) and the critical value ${y_{\mathrm{SS}}}^{H}$ (≅8.58 µM), at which the SS becomes unstable (the Hopf bifurcation at $y_{0}\equiv {y_{0}}^{H}$) determines the minimum amplitude ${C_{\mathrm{ex}}}^{\min}$ of the incoming excitatory pulse that triggers a spike in the A cell at a given *y*_0_. In table 1, we present the data for *y*_SS_ and ${C_{\mathrm{ex}}}^{\min}$ as a function of *y*_0_. The most right column demonstrates that the difference $\left(y_{\mathrm{SS}}-{y_{\mathrm{SS}}}^{H}\right)$ is almost equal to the injected concentration of activator ([Ag^+^]), ${C_{\mathrm{ex}}}^{\min}\Delta t$, per single pulse, if *y*_0_ is far enough from ${y_{0}}^{H}$ (more than 20%). To establish different levels of excitability in different A cells, ${y_{0}}^{\left(m\right)}$ can assume (in general) different values in different *m*th cells.

**Table 1.** The dependences of the steady-state *y*_SS_ and the minimum amplitude ${C_{\mathrm{ex}}}^{\min}$ of a single excitatory incoming pulse on *y*_0_. The amplitude ${C_{\mathrm{ex}}}^{\min}$ should be large enough to generate a spike in an excitable BZ cell described by equations (2.1)–(2.4) plus (2.6) and (2.7) under the condition that the duration of the pulse Δ*t* = 5 s. At chosen parameters and *h* = 0.3 M, ${y_{0}}^{H}≅1.3331\;\mathrm{mM}$ and ${y_{\mathrm{SS}}}^{H}≅8.58\;\text{μ}M$. Data of column ‘$\left(y_{\mathrm{SS}}-{y_{\mathrm{SS}}}^{H})/({C_{\mathrm{ex}}}^{\min}\Delta t\right)$’ are calculated from the values of columns ‘*y*_SS_' and ‘${C_{\mathrm{ex}}}^{\min}$’.

*y*_0_ (mM)	*y*_SS_ (µM)	${C_{\mathrm{ex}}}^{\min}$ (M s^−1^)	$\left(y_{\mathrm{SS}}-{y_{\mathrm{SS}}}^{H})/({C_{\mathrm{ex}}}^{\min}\Delta t\right)$
1.336	8.59	1.08 × 10^−7^	0.019
1.4	8.91	1.79 × 10^−7^	0.37
1.7	10.52	6.10 × 10^−7^	0.64
2.0	12.5	1.07 × 10^−6^	0.73
3.0	18.2	2.58 × 10^−6^	0.75
4.0	23.7	4.03 × 10^−6^	0.76
6.0	35.0	6.81 × 10^−6^	0.78
8.0	46.2	9.53 × 10^−6^	0.79
20.0	113	2.55 × 10^−5^	0.82

In some cases, A cells can be connected by inhibitory pulses to suppress unwanted cells which start oscillating. In this case, equation (2.6) is modified as follows:

2.8 $$\frac{dy_{m}}{dt}=F(x_{m},y_{m},z_{m},v_{m})-k_{\mathrm{diff}}[Ag_{m}]y_{m}+\sum_{n\neq m}\left[{C_{\mathrm{inh}}}^{\left(A\right)}\times P(x_{n},\tau ,\Delta t)\right].$$

Pulsatile periodic external signals are generated by function *P*(*S*(*t*),*τ*,Δ*t*), where

2.9 S(t)=H(sin⁡(ωt)−0.99).

*H*(*x*) is the Heaviside step function. The value of *τ* (which is small) is not important in this case, because only period (=2π/*ω*) and the width of the pulses (=Δ*t*) make sense for periodic perturbation.

## 3. Results

### 3.1. Time delays

Our first method of the recognition of the modes is based on the phase shifts between different oscillators of the CPG and on the polychronization hypothesis [28,29], which states that two pulses from two neurons that generate spikes at different moments of time can come to the third neuron synchronously if time delays Δ*t*_d_ between these two spikes and the difference in the distances between the third neuron and each of these two, Δ*l*, are related as Δ*l*/Δ*t*_d_ = *v*, where *v* is the speed of pulse propagation along axons. Therefore, two conditions should be fulfilled for the satisfactory functioning of the first method: (i) the A cells are tuned in such a way that only four pulses simultaneously coming to the cell can generate a spike in it, (ii) time delays ${\tau_{d}}^{\left(i\right)}$ between the moment of spike of the *i*th CPG oscillator and the moment of pulse arrival to the *m*th A cell are chosen (or tuned) in such a way that all four pulses from the CPG oscillators come simultaneously only to a single A cell that corresponds to the appropriate dynamical mode of the CPG. These ideas remind the methods of computing in networks of spiking neurons with different time delays for different information paths [30].

As an example, consider the AP mode, when oscillators 1 and 3 are in-phase (the first cluster) and oscillators 2 and 4 are in-phase as well (the second cluster) and the two clusters oscillate AP with the global period *T*. Let us select time delays between the CPG oscillators and an arbitrary A cell as follows: ${\tau_{d}}^{\left(1\right)}=\tau_{s}$, ${\tau_{d}}^{\left(2\right)}=\tau_{s}+T/2$, ${\tau_{d}}^{\left(3\right)}=\tau_{s}$, ${\tau_{d}}^{\left(4\right)}=\tau_{s}+T/2$, where time *τ*_s_ is a small arbitrary value (for example, *τ*_s_ = 0.1 *T*). In this case, all four pulses generated by four spikes in the CPG come to the selected A cell simultaneously and induce a spike.

As we mentioned above, there are 26 different modes of the CPG if we take into account permutations. Accordingly, we should have at least 26 A cells, if each cell is responsible for only one mode, or, alternatively, 26 unique combinations of A cells that should indicate the certain modes.

Is it possible to find such a unique set of time delays that only one A cell is excited by a certain sub-mode of the CPG? We answer this question positively and present table 2 with the appropriate sets of time delays for each dynamical sub-mode. All A cells (from 1 to 26) are tuned to the corresponding sub-mode of the CPG due to appropriate sets of ${\tau_{d}}^{\left(i\right)}$. Time delays ${\tau_{d}}^{\left(i\right)}$ are determined by the global period *T* and phase shifts between clusters. All A cells have the same threshold of excitability which is determined by *y*_0_. In our simulations, we take *y*_0_ = 4.0 mM, though other values can be used as well. For the value of *y*_0_ used, the total amplitude of four simultaneous pulses (=the sum of four), ${C_{\mathrm{ex}}}^{\min}$, should exceed 4.03 µM s^−1^ (see line 6 in table 1), while the sum of three simultaneous pulses should be less than 4.03 µM s^−1^. Therefore, for a single unidirectional pulse from the CPG cells to A cells, we use the coupling strength *C*_ex_ ≅ 1.3 µM s^−1^, which is larger than 4.03/4 µM s^−1^ and smaller than 4.03/3 µM s^−1^.

**Table 2.** Time delays ${\tau_{d}}^{\left(i\right)}$ for perturbing pulses from the *i*th CPG oscillator to all A cells. Time shift *τ*_s_ is an arbitrary and small value (≅0.1 *T*).

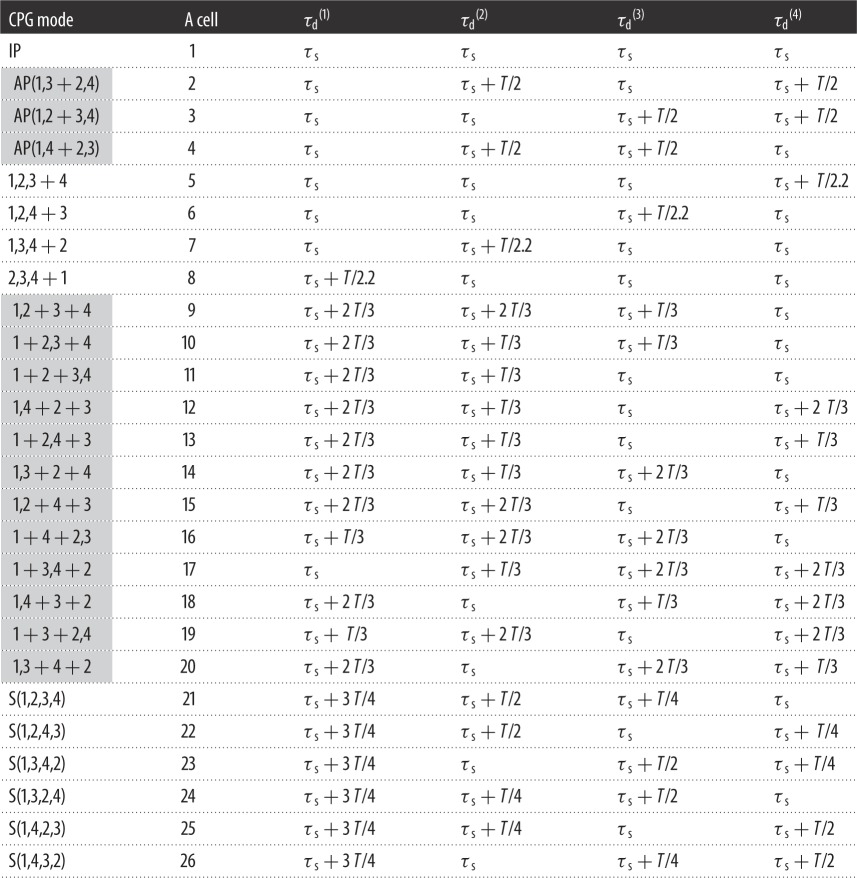

In table 2, we introduce new notations such as ‘(*n,m *+ *k,l*)', ‘(*n,m,l *+ *k*)' or ‘(*n,m + l*+*k*)'. In these notations, such combinations as ‘*n,m,l*' or ‘*n,m*' mean triplet and doublet, respectively, where indices of oscillators (*n, m, k* and *l*) can assume any different integers from 1 to 4. Sign(s) ‘plus' in these notations means a combination of two or three clusters (like doublet + two singlets) separated in time by some phase shift. Notations of the types (1,3 + 2,4) (with commas) and ‘2 + 1 + 1' (without comma) are different: in the first case, we explore the indexes of the oscillators, while in the second case we use the number of oscillators in different clusters.

For the asymmetrical two-cluster modes ‘(*n,m,l *+ *k*)', the difference between appropriate time delays ${\tau_{d}}^{\left(i\right)}$ is equal to *T*/2.2, while for the symmetrical AP mode, the difference between appropriate time delays ${\tau_{d}}^{\left(i\right)}$ is equal to *T*/2.

**Figure 4.**
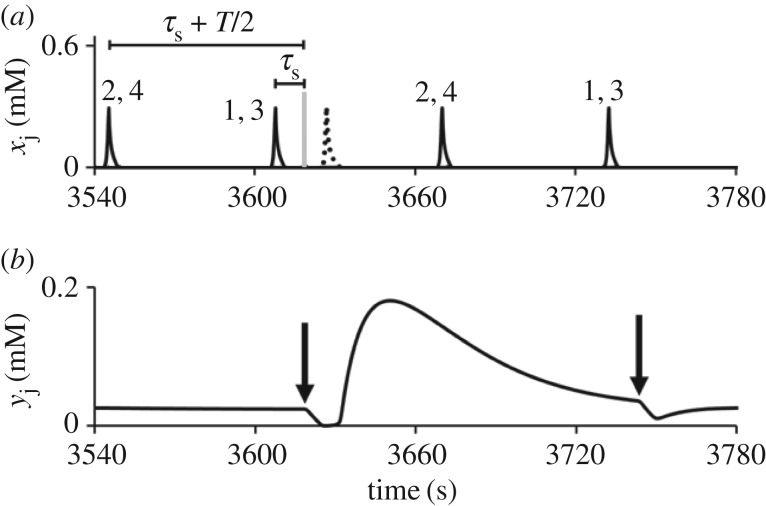
Detection of the AP(1,3 + 2,4) mode of the CPG by A cell at *C*_inh_ = 7 × 10^−5^ M, *τ* = 1 s, *y*_0_ = 4 mM, *C*_ex_ = 1.3 × 10^−6^ M, and ${\tau_{d}}^{\left(1\right)}={\tau_{d}}^{\left(3\right)}=\tau_{s}$, ${\tau_{d}}^{\left(2\right)}={\tau_{d}}^{\left(4\right)}=\tau_{s}+T/2$. (*a*) Spikes of the CPG oscillators (bold lines) and a single spike of the A cell (dotted line); numbers above spikes are indexes of oscillators. Vertical grey line (between spikes of the 1,3 oscillators and the spike in the A cell) mark the moment of time when all four pulses come to the A cell. Vertical arrows in (*b*) mark the same events. (*b*) The dynamics of inhibitor *y* in the A cell.

Simulations of all modes with time delays presented in table 2 confirm that only one A cell is activated in response to a current dynamical mode of the CPG. An example of the detecting of the AP mode (1,3 + 2,4) by the A cell is shown in figure 4. Time delays used for this case are shown in table 2 (the second A cell). As is seen in figure 4*a*, the second A cell generates a spike (dotted line) after receiving four pulses marked by a grey vertical bar. Only this A cell receives four pulses simultaneously. No other A cell is active.

Note that the frequency of spikes induced in the A cell is two times smaller than the frequency of the AP mode in the CPG, which induces these spikes by sending the pulses. In figure 4*b*, we illustrate and explain this phenomenon. After a spike in the A cell, the concentration of inhibitor, *y*, first decreases (down to almost zero), then becomes larger (up to 0.2 mM), and then slowly decreases again. At the moment when the next four pulses come to the A cell simultaneously (marked by the arrows in figure 4*b*), *y* is still large enough for the A cell to become excited. For the task of mode recognition, the fact that different A cells may have different frequencies of spiking activity is not important because we just have to know which A cell is in the oscillatory state. But if one wants to have the same frequencies of the CPG and the A cell, the parameters of the A cell should be changed, for example, *c*_0_ can be decreased or/and [MA] and *h* can be increased.

In real laboratory experiments, periods of all ‘identical' CPG oscillators are slightly different (by a few per cent) [23]. Therefore, we decided to check how a small frequency dispersion of the CPG oscillators affects the response of the A cells. In general, the effect of noise or dispersion of some parameters on the dynamics of the network of coupled oscillators [20] is not within the scope of the present work. We just show that this problem is important demonstrating an example of the functioning of the A cell tuned to detect the IP mode under the condition of frequency dispersion.

To have the CPG oscillators with slightly different natural periods *T*_0*i*_, we explore slightly different parameters *h_i_* (*i* = 1, 2, 3, 4): specifically *h*_1_ = *h*_0_ + *ε*, *h*_2_ = *h*_0_ + *ε*/2, *h*_3_ = *h*_0_ − *ε* and *h*_4_ = *h*_0_ − *ε*/2 at *h*_0_ = 0.3 M and *ε* = 0.01 M. Natural period *T*_0_ of the BZ oscillator (equations (2.1)–(2.4)) almost linearly depends on *h*, *T*_0_ ≅ *ah* + *b*, where *a* = −693.56 s M^−1^ and *b* = 353 s [12,23]. Such dispersion in *T*_0*i*_ leads (for example, for the IP mode) to the fact that ‘synchronous’ spikes are not completely synchronous and there are some small time intervals between them, which are around 1–2 s at the averaged *T*_0_ ≅ 144 s. For the sharp spikes and the standard duration of the pulses Δ*t* = 5 s, even such small asynchrony of the ‘synchronous' spikes leads to the fact that the total amplitude of four pulses is not enough to activate the A cell (see the first line in table 3), which would be activated in the case of completely identical oscillators.

**Table 3.** The response of the A cell tuned for the detection of the IP mode at different *C*_ex_ and the durations of the excitatory pulses Δ*t* under the condition of frequency dispersion related to the differences in *h* (*ε* = 0.01 M) of the CPG oscillators. Parameters of the CPG coupling are *C*_inh_ = 2 × 10^−5^ M s^−1^, *τ* = 30 s.

*C*_ex_ (µM/s)	Δ*t* (s)	C*_ex_Δ*t* (µM)	spike
1.3	5	6.5	−
1.3	7.2	9.36	−
1.3	7.3	9.49	+
1.69	5	8.45	−
1.742	5	8.71	+
13	0.5	6.5	+

To find a possible way to solve the issue, we changed the values of *C*_ex_ and Δ*t* of the excitatory pulses. The result is shown in table 3. As is seen, to activate the A cell, two variants are possible; they are to increase Δ*t* at constant *C*_ex_ (=1.3 µM s^−1^) or to increase *C*_ex_ at Δ*t* = 5 s under the condition that the product *C*_ex_Δ*t* exceeds some critical value. Note that the product *C*_ex_Δ*t* is the amount of the injected activator. It is even possible to find such small Δ*t* and such large *C*_ex_ that the activation of the A cell occurs (see the last line of table 3) at the same *C*_ex_Δ*t* as in the first row of table 3 when the A cell was not activated at Δ*t* = 5 s.

Theory of polychronization [29] works well for two pulses which come to a neuron synchronously. Probability of the event that three or even four pulses come to a neuron synchronously is small and decreases with the number of pulses, especially if neurons are scattered in the space randomly. For the IP, AP and even 3Cl modes of the CPG consisting of four oscillators, this probability being small does not reach zero. However, this probability can be equal to zero for the S mode if, as we supposed at the beginning of this section, the speed *v* of pulse propagation along axons is constant and all axons can be considered as straight segments. Indeed, let us try to find a geometrical point O which is distant from the four vertices *V_i_* of the pyramid at distances *l*, *l* + *vτ*, *l* + 2*vτ*, and *l* + 3*vτ*, where *τ* ≅ *T*/4. The geometric place of points (locus) which are distant from two arbitrary vertices at distances *l* and *l* + *vτ* are the intersection of two spheres with radii *l* and *l* + *vτ*, respectively. Obviously, this locus is a circumference, which is a one-dimensional line. The same is true for the other two vertices of the pyramid. A searched point O should be an intersection of these two circumferences. But two lines may have no mutual points in the three-dimensional space. Therefore, a searched point (i.e. the A cell tuned to detect S modes) may not exist geometrically.

To resolve this problem in future experiments, it would be necessary to introduce intermediate excitable cells (interneurons), which transform the path between CPG oscillators and A cells from the straight to curved line, thus enlarging the distance between the CPG oscillators and A cells and increasing time delays.

### 3.2. Detection of clusters by amplitude

The second method for mode recognition is based on the summation of the excitatory pulses from the CPG oscillators by A cells assuming that the pulse generated by a spike of the CPG oscillator reaches A cell (to which the oscillator is connected) immediately (without delay) or with the same (or almost the same) time delays for all CPG oscillators and A cells. There are three different types of summations for pulses from four CPG oscillators: (i) summation of all four pulses (as in the previous case but without taking into account time delays), (ii) summation of three pulses from any triplet of the four CPG oscillators and (iii) summation of two pulses from any doublet (pairs) of the four CPG oscillators.

For case (i), such a summation makes sense if the thresholds of excitability of A cells are different. Specifically, the first, second and third A cells should be excitable only for four, three (or more) and two (or more) simultaneous pulses, respectively. The fourth A cell should have the minimum threshold that can be overcome by only one pulse. The thresholds of these cells can be named 4P, 3P, 2P and 1P, respectively. Such different values of thresholds can be achieved by using different values of $y_{0}:{y_{0}}^{\left(1\right)}=8\;\mathrm{mM}$, ${y_{0}}^{\left(2\right)}=6\;\mathrm{mM}$, ${y_{0}}^{\left(3\right)}=4\;\mathrm{mM}$, ${y_{0}}^{\left(4\right)}=2\;\mathrm{mM}$) (see also data from table 1). For case (ii), the thresholds of all A cells are the same and correspond to three pulses (3P), while for case (iii), the thresholds of all A cells are the same and correspond to two pulses (2P).

The responses of all A cells described above are presented in table 4 for case (i), in table 5 for case (ii), and in table 6 for case (iii). Signs ‘+’ and ‘−’ in these tables mean that spikes are or are not generated, respectively, in a corresponding A cell. As is seen from table 4, summation of four pulses by four A cells with different levels of excitability can determine the IP mode (by four active A cells), the 2Cl mode of the type ‘1 + 3’ (by three active A cells) and the S mode (by one active A cell). The AP mode and the 3Cl mode of the type ‘1 + 1 + 2’ look identical (two active A cells in both cases), which is a drawback of this method. The other drawback is the fact that any permutations of the ‘1 + 3’ mode (four permutations) or the S mode (six permutations) (see also table 2 for all permutations) cannot be distinguished.

**Table 4.** Summation of simultaneous pulses-spikes from all four CPG oscillators under the condition that the thresholds of four A cells are equal to ‘4 pulses', ‘3 pulses', ‘2 pulses' and ‘1 pulse', respectively.

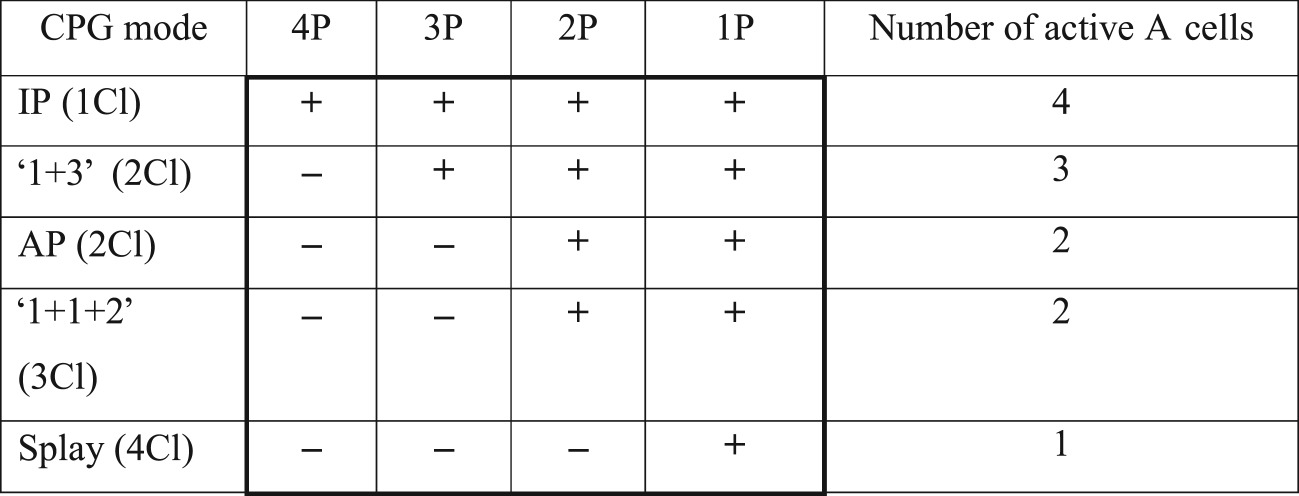

**Table 5.** Summation of simultaneous pulses-spikes from all four groups of three CPG oscillators under the condition that the thresholds of all four A cells of this type are equal to ‘three pulses’. A cell ‘*k,l,m*’ accepts pulses from oscillators *k, l* and *m*, respectively.

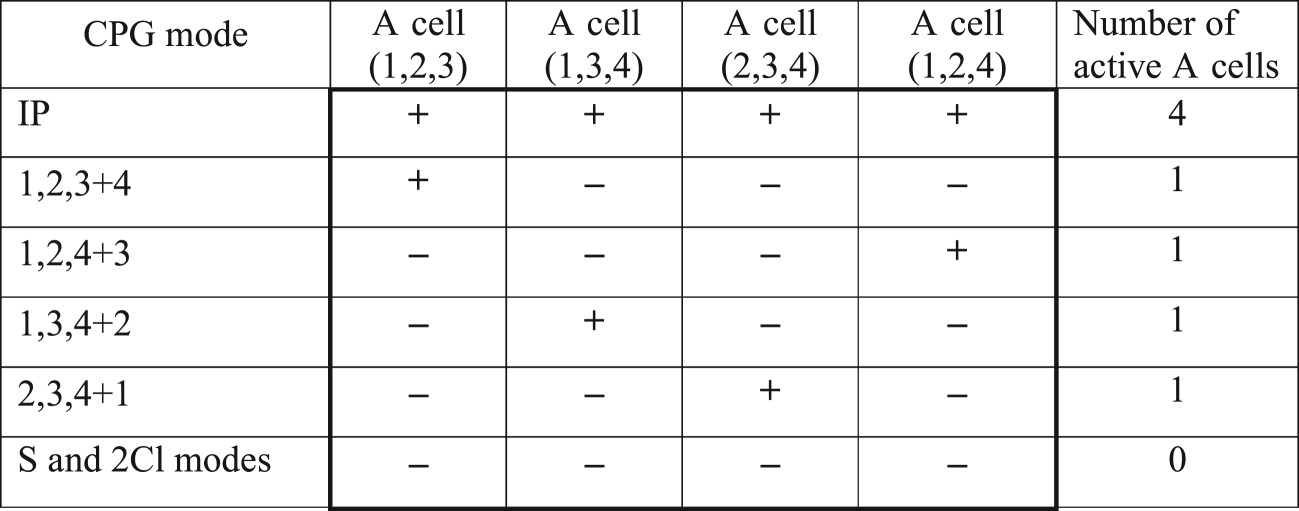

**Table 6.** Summation of simultaneous pulses-spikes from all pairs of oscillators of the CPG under the condition that the threshold of all six A cells is equal to ‘two pulses’. A cell ‘*i,j*’ accepts pulses from oscillators *i* and *j*.

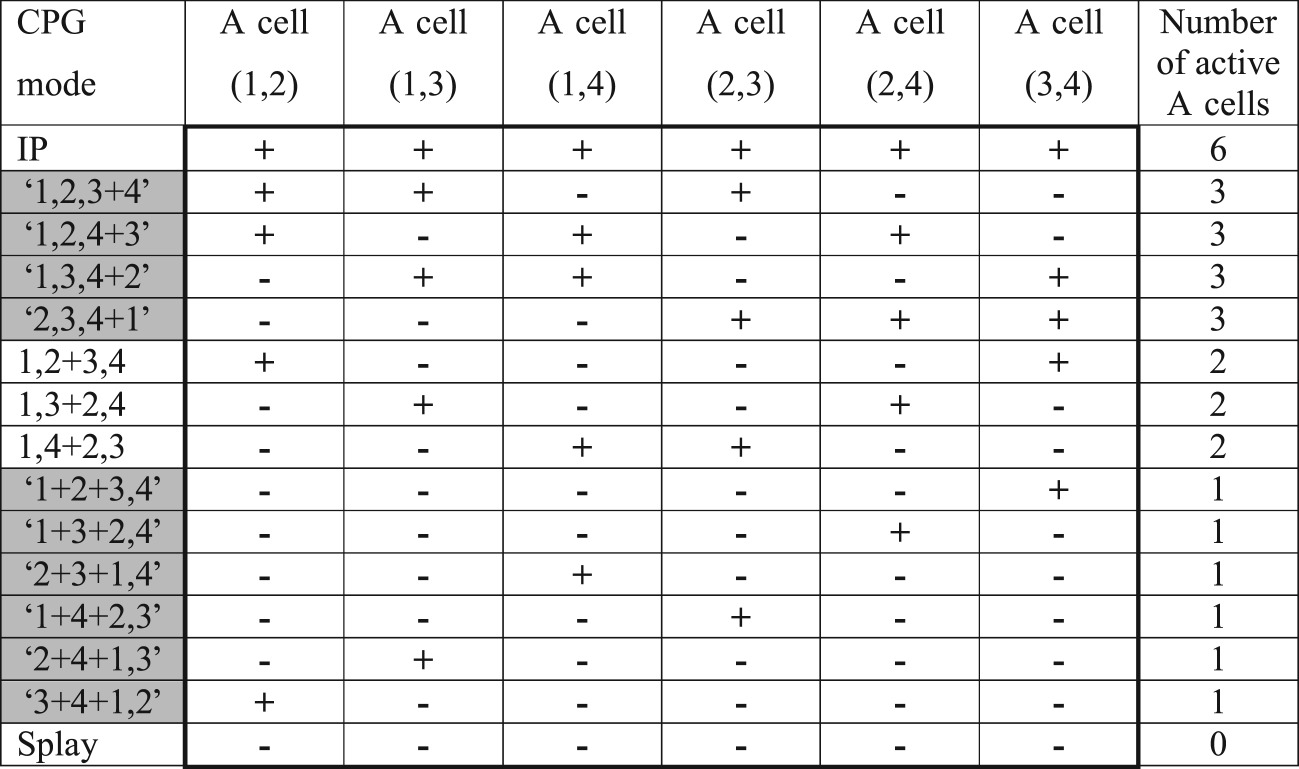

Summation of three pulses in case (ii) can help us to distinguish between all four permutations in the ‘1 + 3' mode. As is seen in table 5, different ‘1 + 3' modes activate different A cells (one A cell per each ‘1 + 3' mode).

Summation of two pulses from all pairs of four CPG oscillators in case (iii) is very effective, as is seen in table 6. The AP mode generates two active A cell, while the 3Cl modes generates only one active A cell. In addition, all permutations inside the AP and 3Cl modes activate different sets of active A cells. Six permutations of 12 for the ‘1 + 1 + 2' (3Cl) mode can also be recorded. But permutations that are different by the sequence of singlet spiking like, for example, ‘1 + 3 + 2,4' and ‘3 + 1 + 2,4' cannot be distinguished.

Different permutations of the S mode cannot be distinguished by the ‘amplitude’ method. ‘Time delay' method should be used in this case.

### 3.3. Resonances

Time intervals between successive spikes of the CPG, Δ (or lag Δ), strongly depend on the dynamical mode of the CPG. For the IP, AP and S modes, Δ is equal approximately to *T*_0_, *T*_0_/2 and *T*_0_/4, respectively. In addition, lag Δ is dependent on the coupling strength *C*_inh_ and time delay *τ* [12,13]. In some sense, the CPG can be considered as a ‘frequency transformer' or ‘frequency generator'. We can use this feature to construct special A cells that could respond resonantly on special frequencies.

**Figure 5.**
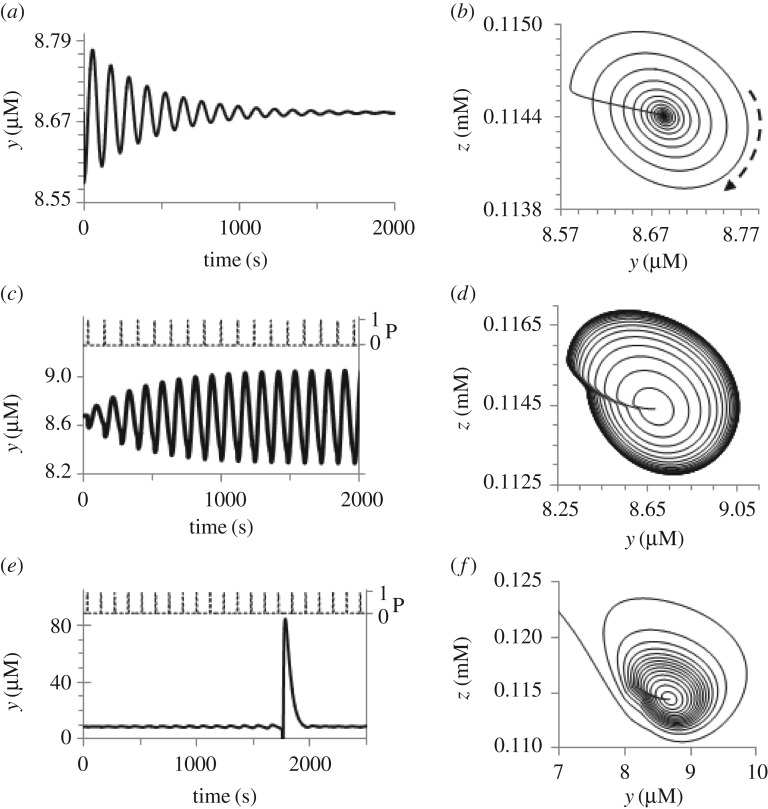
Kinetics of the excitable A cell (*a,c,e*) and the corresponding phase portraits (*b,d,f*) at (*a,b*) initial small perturbation and at periodic pulsatile perturbation *C*_ex_ × *P(S*,*τ*,Δ*t*), (*c,d*) for $C_{\mathrm{ex}}<{C_{\mathrm{ex}}}^{\mathrm{cr}}$ and (*e,f*) for $C_{\mathrm{ex}}>{C_{\mathrm{ex}}}^{\mathrm{cr}}$. Periodic pulses *P* = *P(S*,*τ*,Δ*t*) are shown in panels (*c,e*) (right axis); *S* = *H*(sin(*ω*t)−0.99), *H*(*x*) is the Heaviside function. Parameters of the system (2.1)–(2.4),(2.6),(2.7): *h* = 0.371757 M, *y*_0_ = 1.7743 mM, *ω* = 0.052 s^−1^, *C*_ex_ = (*a*,*b*) 0, (*c*,*d*) 1.3 × 10^−8^ M s^−1^, (*e*,*f*) 1.65 × 10^−8^ M s^−1^; *x*_ini_ = (*a*,*b*) 1.2*x*_SS_; (*c--f*) *x*_SS_; *x*_SS_ = 2.3055 × 10^−7^ M; *y*_ini_ = *y*_SS_ = 8.68402 × 10^−6^ M; *z*_ini_ = *z*_SS_ = 1.14411 × 10^−4^ M; *v*_ini_ = *v*_SS_ = 5.270125 × 10^−4^ M; Im(*λ*) ≅ 0.0538 s^−1^; Im(*λ*)/Re(*λ*) ≅ −22.2.

This method is based on the dynamics of a stable focus steady state (SS) that can respond to a relatively large perturbation in a threshold manner (excitable state). The dynamics of system (2.1)–(2.4) in response to a small perturbation of the SS is described by the following equation (for variable *y*, for example):

3.1 $$y-y_{\mathrm{SS}}=y_{\mathrm{ini}}\cos(\omega_{0}t)\exp(2.3\text{Re}(\lambda )t),$$

where *y*_ini_ is the initial value of *y* immediately after a small pulsed perturbation, *ω*_0_ = Im(*λ*), Im(*λ*) and Re(*λ*) are imaginary and real parts of the largest eigenvalue *λ* of the linearized system (2.1)–(2.4) (see figure 3), Re(*λ*) < 0. A typical dynamics of system (2.1)–(2.4) in response to such small perturbation of the stable focus is shown in figure 5*a*,*b*.

If the pulsatile periodic external signal *C*_ex_ × *P*(*S*,*τ*,Δ*t*) (see equation (3.1)) has the frequency *ω* which is close to *ω*_0_, a resonance should be observed, i.e. a spike should be generated at a relatively small amplitude *C*_ex_ (but larger than some critical value ${C_{\mathrm{ex}}}^{\mathrm{cr}}$) after a series of pulsed perturbations. In figure 5*c*,*d*, the dynamics of the excitable A cell at $C_{\mathrm{ex}}<{C_{\mathrm{ex}}}^{\mathrm{cr}}$ is exhibited. As is seen, the system departs from the SS at initial pulses, but then reaches some stable trajectory around the SS, which is similar (in some sense) to attractor. In figure 5*e*,*f*, when ${C_{\mathrm{ex}}}^{\mathrm{cr}}<C_{\mathrm{ex}}$, the trajectory of the system in the phase space leaves the basin of attraction of the SS, and a high-amplitude spike is generated. After this spike, the system tries to return to the SS, but periodic perturbation will generate the next spike after several periods of the external signal.

**Figure 6.**
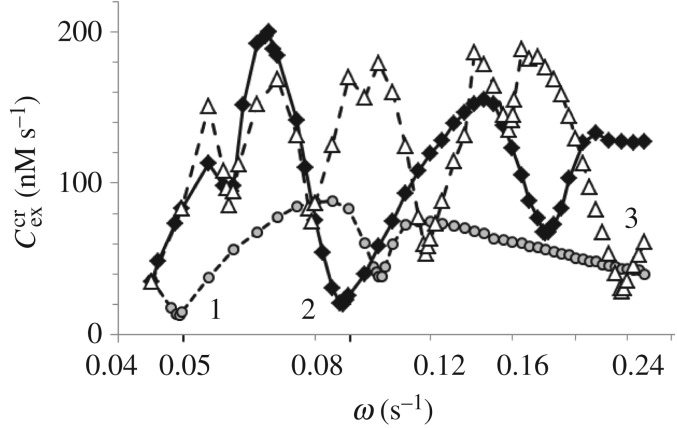
Resonance responses of three A cells on external periodic pulsatile signal *C*_ex_ × *P*(*ω*) with frequency *ω* and amplitude *C*_ex_. Three regular modes, IP, AP and splay, can be detected by three A cells with the response curves 1, 2 and 3, respectively. If $C_{\mathrm{ex}}<{C_{\mathrm{ex}}}^{\mathrm{cr}}$, the A cell remains in the vicinity of the steady state. Parameters of the A cells and corresponding eigenvalues of the linearized system (2.1)–(2.4): *h*/M = (curve 1) 0.3588, (curve 2) 0.4966, (curve 3) 0.8623; *y*_0_/M = (1) 0.001697, (2) 0.002567, (3) 0.005429; Im(*λ*) = (1) 0.05053, (2) 0.09044, (3) 0.24571; Im(*λ*)/Re(*λ*) = (1) 21.55, (2) 21.56, (3) 21.74. Axis ‘*ω*’ is logarithmic.

The value of ${C_{\mathrm{ex}}}^{\mathrm{cr}}$ strongly depends on *ω* and parameters of the system, i.e. on the proximity of the system to the Hopf bifurcation and on the ratio Im(*λ*)/Re(*λ*). Typical dependences of the ${C_{\mathrm{ex}}}^{\mathrm{cr}}$ on *ω* for three different values of *ω*_0_ [=Im(*λ*)] and at approximately constant Im(*λ*)/Re(*λ*) (≅21.6) is presented in figure 6. As expected (because these curves are analogous to the Arnold tongue), each curve has the minimum of ${C_{\mathrm{ex}}}^{\mathrm{cr}}$ at *ω* ≅ *ω*_0_. In addition, local minima are observed in figure 6 at *ω* ≅ 2*ω*_0_ (for curves 1 and 2) and *ω* ≅ *ω*_0_/2 and *ω* ≅ *ω*_0_/3 (for curve 3).

To use three A cells to detect IP, AP and S modes, respectively, we should tune the response curves of the A cells to the frequencies of these modes of the CPG. The A cells with the response curves 1, 2 and 3 shown in figure 6 are already tuned to the frequencies ${\omega_{0}}^{\left(m\right)}=2\pi n/T_{m}$, where *m* = *n* = 1 for the IP mode, *m* = *n* = 2 for the AP mode and *m* = 3 and *n* = 4 for the S mode, *T_m_* is the global period of the corresponding mode obtained at the following parameters of the CPG: *h* = 0.3 M, *C*_inh_ = 2 × 10^−5^ M s^−1^ and *τ* = 40 s for the IP mode; *h* = 0.3 M, *C*_inh_ = 2 × 10^−5^ M s^−1^ and *τ* = 1 s for the AP mode; *h* = 0.29 M, *C*_inh_ = 10^−4^ M s^−1^ and *τ* = 60 s for the S mode. For these modes, ${\omega_{0}}^{\left(1\right)}=0.0506\;s^{-1}$, ${\omega_{0}}^{\left(2\right)}=0.0962\;s^{-1}$ and ${\omega_{0}}^{\left(3\right)}=0.2349\;s^{-1}$. Note that frequencies ${\omega_{0}}^{\left(m\right)}$ are very close (almost equal) to the values of Im(*λ*) and to the frequency of the minima of the corresponding curves in figure 6.

In our computer experiment, all three A cells (A1 for the IP mode, A2 for the AP mode and A3 for the S mode) receive excitatory pulses from four oscillators of the CPG immediately after the spikes in these cells. The amplitudes ${C_{\mathrm{ex}}}^{\left(m\right)}$ of these pulses are selected in the following way. For the A1 cell (which is intended to detect the IP mode), ${C_{\mathrm{ex}}}^{\left(1\right)}$ is just slightly larger than the minimum value ${C_{\mathrm{ex}}}^{\mathrm{cr}}/4$ for curve 1 at ${\omega_{0}}^{\left(1\right)}≅0.05\;s^{-1}$, but smaller than ${C_{\mathrm{ex}}}^{\mathrm{cr}}/4$ of the other curves at the same *ω*; ${C_{\mathrm{ex}}}^{\left(1\right)}≅6\;\mathrm{nM}\;s^{-1}$. For the A2 cell (AP mode), ${C_{\mathrm{ex}}}^{\left(2\right)}$ is larger than the minimum value ${C_{\mathrm{ex}}}^{\mathrm{cr}}/2$ for curve 2 at ${\omega_{0}}^{\left(2\right)}≅0.095\;s^{-1}$, but smaller than ${C_{\mathrm{ex}}}^{\mathrm{cr}}/2$ of the other curves at the same *ω*; the value of ${C_{\mathrm{ex}}}^{\left(2\right)}=25\;\mathrm{nM}\;s^{-1}$ works well. For the A3 cell (S mode), ${C_{\mathrm{ex}}}^{\left(3\right)}>{C_{\mathrm{ex}}}^{\mathrm{cr}}$ for curve 3 at ${\omega_{0}}^{\left(3\right)}≅0.23\;s^{-1}$, but smaller than ${C_{\mathrm{ex}}}^{\mathrm{cr}}$ of the other curves at the same frequency; ${C_{\mathrm{ex}}}^{\left(3\right)}=29\;\mathrm{nM}\;s^{-1}$. Simulations demonstrate that only one A cell responds to the corresponding mode.

The values of the resonance frequencies ${\omega_{0}}^{\left(m\right)}$ and the minimum values of ${C_{\mathrm{ex}}}^{\mathrm{cr}}$ at ${\omega_{0}}^{\left(m\right)}$ can be tuned in a broad range just varying such parameters as *y*_0_ and/or *h* for the A cell: the larger the ratio Im(*λ*)/Re(*λ*), the smaller the minimum value of ${C_{\mathrm{ex}}}^{\mathrm{cr}}$ and the more pronounced is the resonance. The frequencies of the responding A cells depend on the amplitudes ${C_{\mathrm{ex}}}^{\left(m\right)}$: the larger the ${C_{\mathrm{ex}}}^{\left(m\right)}$, the larger the frequency, which varies in our case in the range $(0.2\text{--}0.5)\;{\omega_{0}}^{\left(i\right)}$.

## 4. Discussion

Between external signals S and the ‘heart' of the neural network, the CPG, we place two subsystems, the analysing unit (a ‘reader') and the decision-making (DM) unit. These two subsystems separate an environment (which is S) and a responsive specimen (which is the CPG). We think that such architecture of the neural network (which includes feedbacks) promotes a smart (or adaptive) behavior of the entire organism (neural network). Our architecture can be considered as development of the ideas for heteroclinic computing [19,20,31–33].

We suggested three general methods for mode recognition: ‘time delays', the amplitude method for cluster identification and the resonance method. All these methods can be combined and work together compensating some drawbacks of the other methods or fulfilling different tasks. For example, the resonance method can determine patterns with different frequencies using just a few A cells, but cannot distinguish patterns of permutation or patterns with close frequencies like, for example, AP and ‘3 + 1' modes. On the other hand, the amplitude method requires more A cells. In the most sensitive version of this method, when A cells are tuned to detect doublets, two A cells are responding to a single AP mode, three A cells are responding to the triplet–singlet modes, and all six A cells respond to the IP mode. If the DM unit is built in such a way that it can recognize different modes by an appropriate combination of activated A cells, then no additional improvements of the A block are required. But if the DM unit works properly only in the case when only one specific A cell is activated, then we should add an additional ‘filter’ or additional layer of A cells to resolve the issue. Obviously, it can be done in many ways, including implementation of additional inhibitory coupling between A cells or addition of other A cells in the next layer that collect signals from the A cells in the first layer. This is mostly due to an engineering problem that could be resolved after constructing the DM unit.

The methods of mode recognition suggested in the present work can be easily extended for the CPG consisting of five, six or more oscillators. The functional organization of large circuits can be based on the existing knowledge of small circuits [2]. Since we considered very general physical methods of mode recognition, the analogous principals of the ‘reading system' might be found in the brain.

To construct the first chemical computer we are planning to develop the DM unit in the nearest future. Logic elements or even fuzzy logic [34] should be employed inside this block, especially if we take into account that the logic gate was developed recently using the BZ cells [14,15]. Experimental verification of the A unit in cooperation with the chemical CPG unit is in progress.

## Supplementary Material

S1. The FlexPDE script

## Supplementary Material

S2. The FlexPDE script

## Supplementary Material

S3. The FlexPDE script

## Supplementary Material

S4. The FlexPDE script

## Supplementary Material

S5. The FlexPDE script

## Data accessibility

Mathematical codes (The FlexPDE scripts) supporting the results for each method described in this study are available as electronic supplementary material: S1–S5.
