# Supplementary material for: A ‘reader’ unit of the chemical computer
Source: R Soc Open Sci. 2018 Jan 10;5(1):171495. doi: 10.1098/rsos.171495 (PMC5792929; doi:10.1098/rsos.171495)
Supplement: S2. The FlexPDE script [file rsos171495supp2.docx]

TITLE

'ODE'

SELECT

ngrid=1

changelim=0.1

ERRLIM= 0.01

COORDINATES

cartesian1

VARIABLES

Act1

Inh1

Cat1

v1

Act2

Inh2

Cat2

v2

Act3

Inh3

Cat3

v3

Act4

Inh4

Cat4

v4

ActSig1

InhSig1

CatSig1

vSig1

AgSig1(threshold = 0.01)

ActSig2

InhSig2

CatSig2

vSig2

AgSig2(threshold = 0.01)

ActSig3

InhSig3

CatSig3

vSig3

AgSig3(threshold = 0.01)

ActSig4

InhSig4

CatSig4

vSig4

AgSig4(threshold = 0.01)

bs1 (threshold = 0.01)

Pr1 (threshold = 0.01)

bs2 (threshold = 0.01)

Pr2 (threshold = 0.01)

bs3 (threshold = 0.01)

Pr3 (threshold = 0.01)

bs4 (threshold = 0.01)

Pr4 (threshold = 0.01)

bss1 (threshold = 0.01)

Pd1 (threshold = 0.01)

bss2 (threshold = 0.01)

Pd2 (threshold = 0.01)

bss3 (threshold = 0.01)

Pd3 (threshold = 0.01)

bss4 (threshold = 0.01)

Pd4 (threshold = 0.01)

bsSig11 (threshold = 0.01)

PrSig11 (threshold = 0.01)

bsSig12 (threshold = 0.01)

PrSig12 (threshold = 0.01)

bsSig13 (threshold = 0.01)

PrSig13 (threshold = 0.01)

bsSig14 (threshold = 0.01)

PrSig14 (threshold = 0.01)

bsSig21 (threshold = 0.01)

PrSig21 (threshold = 0.01)

bsSig22 (threshold = 0.01)

PrSig22 (threshold = 0.01)

bsSig23 (threshold = 0.01)

PrSig23 (threshold = 0.01)

bsSig24 (threshold = 0.01)

PrSig24 (threshold = 0.01)

bsSig25 (threshold = 0.01)

PrSig25 (threshold = 0.01)

bsSig31 (threshold = 0.01)

PrSig31 (threshold = 0.01)

bsSig32 (threshold = 0.01)

PrSig32 (threshold = 0.01)

bsSig33 (threshold = 0.01)

PrSig33 (threshold = 0.01)

bsSig34 (threshold = 0.01)

PrSig34 (threshold = 0.01)

bsSig35 (threshold = 0.01)

PrSig35 (threshold = 0.01)

bsSig36 (threshold = 0.01)

PrSig36 (threshold = 0.01)

bsSig41 (threshold = 0.01)

PrSig41 (threshold = 0.01)

bsSig42 (threshold = 0.01)

PrSig42 (threshold = 0.01)

bsSig43 (threshold = 0.01)

PrSig43 (threshold = 0.01)

bsSig44 (threshold = 0.01)

PrSig44 (threshold = 0.01)

bsSig45 (threshold = 0.01)

PrSig45 (threshold = 0.01)

bsSig46 (threshold = 0.01)

PrSig46 (threshold = 0.01)

bsSig47 (threshold = 0.01)

PrSig47 (threshold = 0.01)

bssSig11 (threshold = 0.01)

PdSig11 (threshold = 0.01)

bssSig12 (threshold = 0.01)

PdSig12 (threshold = 0.01)

bssSig13 (threshold = 0.01)

PdSig13 (threshold = 0.01)

bssSig14 (threshold = 0.01)

PdSig14 (threshold = 0.01)

bssSig21 (threshold = 0.01)

PdSig21 (threshold = 0.01)

bssSig22 (threshold = 0.01)

PdSig22 (threshold = 0.01)

bssSig23 (threshold = 0.01)

PdSig23 (threshold = 0.01)

bssSig24 (threshold = 0.01)

PdSig24 (threshold = 0.01)

bssSig25 (threshold = 0.01)

PdSig25 (threshold = 0.01)

bssSig31 (threshold = 0.01)

PdSig31 (threshold = 0.01)

bssSig32 (threshold = 0.01)

PdSig32 (threshold = 0.01)

bssSig33 (threshold = 0.01)

PdSig33 (threshold = 0.01)

bssSig34 (threshold = 0.01)

PdSig34 (threshold = 0.01)

bssSig35 (threshold = 0.01)

PdSig35 (threshold = 0.01)

bssSig36 (threshold = 0.01)

PdSig36 (threshold = 0.01)

bssSig41 (threshold = 0.01)

PdSig41 (threshold = 0.01)

bssSig42 (threshold = 0.01)

PdSig42 (threshold = 0.01)

bssSig43 (threshold = 0.01)

PdSig43 (threshold = 0.01)

bssSig44 (threshold = 0.01)

PdSig44 (threshold = 0.01)

bssSig45 (threshold = 0.01)

PdSig45 (threshold = 0.01)

bssSig46 (threshold = 0.01)

PdSig46 (threshold = 0.01)

bssSig47 (threshold = 0.01)

PdSig47 (threshold = 0.01)

DEFINITIONS

h0 = 0.3

heps = 0.00

h1 = h0 + heps

h2 = h0 - heps

h3 = h0 + heps/2

h4 = h0 - heps/2

h5 = h0

A = 0.25

MA = 0.1

CF = 1e-3

k0 = 5e-4

bsr1 = 0.5

bsr2 = 1

b1 = 3

b2 = 3

k11 = 2.00E+06*h1

k12 = 2.00E+06*h2

k13 = 2.00E+06*h3

k14 = 2.00E+06*h4

k15 = 2.00E+06*h5

k21 = 2*h1*h1*A

k22 = 2*h2*h2*A

k23 = 2*h3*h3*A

k24 = 2*h4*h4*A

k25 = 2*h5*h5*A

k3 = 3000

k41 = 42*h1*A

k42 = 42*h2*A

k43 = 42*h3*A

k44 = 42*h4*A

k45 = 42*h5*A

kr = 2E+08

kred = 5E+06

k9p = 20

k10p = 0.05

k10 = k10p*MA

k1_3 = 0.004

cmin =sqrt(3*kr* k10*CF)/kred

time_ini = 0

time_end = 1300

actspike = 1e-4

Cinh = 2e-5

CinhSig = 2e-2

Cact = 2.5e-6

Inh001 = 8e-3

Inh002 = 6e-3

Inh003 = 4e-3

Inh004 = 2e-3

kdiff = 1e8

delta_t = 5

tau_d = 50 !AP = 1; IP = 40, 50; W = 50; Wr = 20, 30; 3Cl = 7 (a-t-a); 2Cl = 8 (a-t-a); - for CPG

period1 = 1 !AP = 123, IP = 114.5, W = 99.8, Wr = 112.8, 3Cl = 160.3, 2Cl = 158

period2 = period1

period3 = period1

period4 = period1

tau_d_coeff11 = 0.1

tau_d_coeff12 = 0.1

tau_d_coeff13 = 0.1

tau_d_coeff14 = 0.1

tau_d_coeff21 = 0.1

tau_d_coeff22 = 0.1

tau_d_coeff23 = 0.1

tau_d_coeff24 = 0.1

tau_d_coeff25 = 0.1

tau_d_coeff31 = 0.1

tau_d_coeff32 = 0.1

tau_d_coeff33 = 0.1

tau_d_coeff34 = 0.1

tau_d_coeff35 = 0.1

tau_d_coeff36 = 0.1

tau_d_coeff41 = 0.1

tau_d_coeff42 = 0.1

tau_d_coeff43 = 0.1

tau_d_coeff44 = 0.1

tau_d_coeff45 = 0.1

tau_d_coeff46 = 0.1

tau_d_coeff47 = 0.1

Qr1 = IF bs1 > 0.5 THEN 0 ELSE 5*delta_t

Qr2 = IF bs2 > 0.5 THEN 0 ELSE 5*delta_t

Qr3 = IF bs3 > 0.5 THEN 0 ELSE 5*delta_t

Qr4 = IF bs4 > 0.5 THEN 0 ELSE 5*delta_t

Qd1 = IF bss1 > 0.5 THEN 0 ELSE 5*tau_d

Qd2 = IF bss2 > 0.5 THEN 0 ELSE 5*tau_d

Qd3 = IF bss3 > 0.5 THEN 0 ELSE 5*tau_d

Qd4 = IF bss4 > 0.5 THEN 0 ELSE 5*tau_d

Qbs1 = IF bs1 > 0.5 THEN 0 ELSE 0.5

Qbs2 = IF bs2 > 0.5 THEN 0 ELSE 0.5

Qbs3 = IF bs3 > 0.5 THEN 0 ELSE 0.5

Qbs4 = IF bs4 > 0.5 THEN 0 ELSE 0.5

QrSig11 = IF bsSig11 > 0.5 THEN 0 ELSE 5*delta_t

QrSig12 = IF bsSig12 > 0.5 THEN 0 ELSE 5*delta_t

QrSig13 = IF bsSig13 > 0.5 THEN 0 ELSE 5*delta_t

QrSig14 = IF bsSig14 > 0.5 THEN 0 ELSE 5*delta_t

QdSig11 = IF bssSig11 > 0.5 THEN 0 ELSE 5*tau_d_coeff11*period1

QdSig12 = IF bssSig12 > 0.5 THEN 0 ELSE 5*tau_d_coeff12*period2

QdSig13 = IF bssSig13 > 0.5 THEN 0 ELSE 5*tau_d_coeff13*period3

QdSig14 = IF bssSig14 > 0.5 THEN 0 ELSE 5*tau_d_coeff14*period4

QbsSig11 = IF bsSig11 > 0.5 THEN 0 ELSE 0.5

QbsSig12 = IF bsSig12 > 0.5 THEN 0 ELSE 0.5

QbsSig13 = IF bsSig13 > 0.5 THEN 0 ELSE 0.5

QbsSig14 = IF bsSig14 > 0.5 THEN 0 ELSE 0.5

QrSig21 = IF bsSig21 > 0.5 THEN 0 ELSE 5*delta_t

QrSig22 = IF bsSig22 > 0.5 THEN 0 ELSE 5*delta_t

QrSig23 = IF bsSig23 > 0.5 THEN 0 ELSE 5*delta_t

QrSig24 = IF bsSig24 > 0.5 THEN 0 ELSE 5*delta_t

QrSig25 = IF bsSig25 > 0.5 THEN 0 ELSE 5*delta_t

QdSig21 = IF bssSig21 > 0.5 THEN 0 ELSE 5*tau_d_coeff21*period1

QdSig22 = IF bssSig22 > 0.5 THEN 0 ELSE 5*tau_d_coeff22*period2

QdSig23 = IF bssSig23 > 0.5 THEN 0 ELSE 5*tau_d_coeff23*period3

QdSig24 = IF bssSig24 > 0.5 THEN 0 ELSE 5*tau_d_coeff24*period4

QdSig25 = IF bssSig25 > 0.5 THEN 0 ELSE 5*tau_d_coeff25*period4

QbsSig21 = IF bsSig21 > 0.5 THEN 0 ELSE 0.5

QbsSig22 = IF bsSig22 > 0.5 THEN 0 ELSE 0.5

QbsSig23 = IF bsSig23 > 0.5 THEN 0 ELSE 0.5

QbsSig24 = IF bsSig24 > 0.5 THEN 0 ELSE 0.5

QbsSig25 = IF bsSig25 > 0.5 THEN 0 ELSE 0.5

QrSig31 = IF bsSig31 > 0.5 THEN 0 ELSE 5*delta_t

QrSig32 = IF bsSig32 > 0.5 THEN 0 ELSE 5*delta_t

QrSig33 = IF bsSig33 > 0.5 THEN 0 ELSE 5*delta_t

QrSig34 = IF bsSig34 > 0.5 THEN 0 ELSE 5*delta_t

QrSig35 = IF bsSig35 > 0.5 THEN 0 ELSE 5*delta_t

QrSig36 = IF bsSig36 > 0.5 THEN 0 ELSE 5*delta_t

QdSig31 = IF bssSig31 > 0.5 THEN 0 ELSE 5*tau_d_coeff31*period1

QdSig32 = IF bssSig32 > 0.5 THEN 0 ELSE 5*tau_d_coeff32*period2

QdSig33 = IF bssSig33 > 0.5 THEN 0 ELSE 5*tau_d_coeff33*period3

QdSig34 = IF bssSig34 > 0.5 THEN 0 ELSE 5*tau_d_coeff34*period4

QdSig35 = IF bssSig35 > 0.5 THEN 0 ELSE 5*tau_d_coeff35*period4

QdSig36 = IF bssSig36 > 0.5 THEN 0 ELSE 5*tau_d_coeff36*period4

QbsSig31 = IF bsSig31 > 0.5 THEN 0 ELSE 0.5

QbsSig32 = IF bsSig32 > 0.5 THEN 0 ELSE 0.5

QbsSig33 = IF bsSig33 > 0.5 THEN 0 ELSE 0.5

QbsSig34 = IF bsSig34 > 0.5 THEN 0 ELSE 0.5

QbsSig35 = IF bsSig35 > 0.5 THEN 0 ELSE 0.5

QbsSig36 = IF bsSig36 > 0.5 THEN 0 ELSE 0.5

QrSig41 = IF bsSig41 > 0.5 THEN 0 ELSE 5*delta_t

QrSig42 = IF bsSig42 > 0.5 THEN 0 ELSE 5*delta_t

QrSig43 = IF bsSig43 > 0.5 THEN 0 ELSE 5*delta_t

QrSig44 = IF bsSig44 > 0.5 THEN 0 ELSE 5*delta_t

QrSig45 = IF bsSig45 > 0.5 THEN 0 ELSE 5*delta_t

QrSig46 = IF bsSig46 > 0.5 THEN 0 ELSE 5*delta_t

QrSig47 = IF bsSig47 > 0.5 THEN 0 ELSE 5*delta_t

QdSig41 = IF bssSig41 > 0.5 THEN 0 ELSE 5*tau_d_coeff41*period1

QdSig42 = IF bssSig42 > 0.5 THEN 0 ELSE 5*tau_d_coeff42*period2

QdSig43 = IF bssSig43 > 0.5 THEN 0 ELSE 5*tau_d_coeff43*period3

QdSig44 = IF bssSig44 > 0.5 THEN 0 ELSE 5*tau_d_coeff44*period4

QdSig45 = IF bssSig45 > 0.5 THEN 0 ELSE 5*tau_d_coeff45*period4

QdSig46 = IF bssSig46 > 0.5 THEN 0 ELSE 5*tau_d_coeff46*period4

QdSig47 = IF bssSig47 > 0.5 THEN 0 ELSE 5*tau_d_coeff47*period4

QbsSig41 = IF bsSig41 > 0.5 THEN 0 ELSE 0.5

QbsSig42 = IF bsSig42 > 0.5 THEN 0 ELSE 0.5

QbsSig43 = IF bsSig43 > 0.5 THEN 0 ELSE 0.5

QbsSig44 = IF bsSig44 > 0.5 THEN 0 ELSE 0.5

QbsSig45 = IF bsSig45 > 0.5 THEN 0 ELSE 0.5

QbsSig46 = IF bsSig46 > 0.5 THEN 0 ELSE 0.5

QbsSig47 = IF bsSig47 > 0.5 THEN 0 ELSE 0.5

{initial conditions}

{1 AP }

{ oscs_path="1 AP 4oscs\"

Act10 = 7.1e-7

Inh10 =1e-7

Cat10 = 3.29342E-5

v10 = 1e-7

Act20 =7.76e-8

Inh20 = 1.12e-7

Cat20 = 1.2e-4

v20 = 1.36e-3

Act30 = 7.1e-7

Inh30 =1e-7

Cat30 = 3.29342E-5

v30 = 1e-7

Act40 = 7.76e-8

Inh40 = 1.12e-7

Cat40 = 1.2e-4

v40 = 1.36e-3

ActSig1_0 = 7.76e-8

InhSig1_0 = 1.12e-7

CatSig1_0 = 1.2e-4

vSig1_0 = 1.36e-3

AgSig1_0 = 0 }

{2 IP -> 1+234 Cinh = 2e-5 tau_d = 8 a-t-a}

{ oscs_path="2 IP 4oscs\"

Act10 = 0.000120394851493317

Inh10 = 2.73192731536462E-7

Cat10 = 0.000302666706771218

v10 = 0.00456601011752023

Act20 = 2.11007022809961E-7

Inh20 = 9.17926951929157E-6

Cat20 = 1.38574910923001E-5

v20 = 0.0045775865331391

Act30 = 3.73131772272833E-7

Inh30 = 6.62729773256394E-6

Cat30 = 1.6877527273958E-5

v30 = 0.00459045122483998

Act40 = 2.43539283655869E-7

Inh40 = 8.34217616499501E-6

Cat40 = 1.45023881912688E-5

v40 = 0.00458774010992285

ActSig1_0 = 7.76e-8

InhSig1_0 = 1.12e-7

CatSig1_0 = 1.2e-4

vSig1_0 = 1.36e-3

AgSig1_0 = 0 }

{2 IP -> 2+ 134 Cinh = 2e-5 tau_d = 8 a-t-a}

{ oscs_path="2 IP 4oscs\"

Act10 = 7.1e-7

Inh10 =1e-7

Cat10 = 3.29342E-5

v10 = 1e-7

Act20 = 7.1e-7

Inh20 =1e-7

Cat20 = 3.29342E-5

v20 = 1e-7

Act30 = 7.1e-7

Inh30 =1e-7

Cat30 = 3.29342E-5

v30 = 1e-7

Act40 = 7.1e-7

Inh40 =1e-7

Cat40 = 3.29342E-5

v40 = 1e-7

ActSig1_0 = 7.76e-8

InhSig1_0 = 1.12e-7

CatSig1_0 = 1.2e-4

vSig1_0 = 1.36e-3

AgSig1_0 = 0 }

{3 W }

oscs_path="3 W 4oscs\"

Act10 = 7.71776479613043E-8

Inh10 = 0.000142379639466751

Cat10 = 0.000254305487778619

v10 = 0.00167433504170452

Act20 = 7.85113049354742E-8

Inh20 = 0.000136040354581379

Cat20 = 0.000783185715106246

v20 = 0.00160135135598607

Act30 = 9.97378691809549E-8

Inh30 = 2.19073279666893E-5

Cat30 = 3.36333291303531E-5

v30 = 0.00154833441905765

Act40 = 8.14222138745477E-8

Inh40 = 5.4966577209892E-5

Cat40 = 9.31964326125288E-5

v40 = 0.00166132632155534

ActSig1_0 = 7.76e-8

InhSig1_0 = 1.12e-7

CatSig1_0 = 1.2e-4

vSig1_0 = 1.36e-3

AgSig1_0 = 0

{4 Wr }

{ oscs_path="4 Wr 4oscs\"

Act10 = 8.14222138745477E-8

Inh10 = 5.4966577209892E-5

Cat10 = 9.31964326125288E-5

v10 = 0.00166132632155534

Act20 = 9.97378691809549E-8

Inh20 = 2.19073279666893E-5

Cat20 = 3.36333291303531E-5

v20 = 0.00154833441905765

Act30 = 7.85113049354742E-8

Inh30 = 0.000136040354581379

Cat30 = 0.000783185715106246

v30 = 0.00160135135598607

Act40 = 7.71776479613043E-8

Inh40 = 0.000142379639466751

Cat40 = 0.000254305487778619

v40 = 0.00167433504170452

ActSig1_0 = 7.76e-8

InhSig1_0 = 1.12e-7

CatSig1_0 = 1.2e-4

vSig1_0 = 1.36e-3

AgSig1_0 = 0 }

{5 12+3+4 }

{ oscs_path="5 12+3+4 4oscs\"

Act10 = 7.7724798e-8

Inh10 = 2.6997116e-4

Cat10 = 5.5233108e-4

v10 = 2.0169811e-3

Act20 = 7.5344063e-8

Inh20 = 2.4148942e-4

Cat20 = 5.9849645e-4

v20 = 1.9673747e-3

Act30 = 7.7893979e-8

Inh30 = 1.1474412e-4

Cat30 = 1.8081622e-5

v30 = 2.080358e-3

Act40 = 7.8181185e-8

Inh40 = 1.5852019e-4

Cat40 = 7.4330636e-5

v40 = 2.1952973e-3

ActSig1_0 = 7.76e-8

InhSig1_0 = 1.12e-7

CatSig1_0 = 1.2e-4

vSig1_0 = 1.36e-3

AgSig1_0 = 0 }

{6 1+23+4 }

{ oscs_path="6 1+23+4 4oscs\"

Act10 = 7.8181185e-8

Inh10 = 1.5852019e-4

Cat10 = 7.4330636e-5

v10 = 2.1952973e-3

Act20 = 7.7724798e-8

Inh20 = 2.6997116e-4

Cat20 = 5.5233108e-4

v20 = 2.0169811e-3

Act30 = 7.5344063e-8

Inh30 = 2.4148942e-4

Cat30 = 5.9849645e-4

v30 = 1.9673747e-3

Act40 = 7.7893979e-8

Inh40 = 1.1474412e-4

Cat40 = 1.8081622e-5

v40 = 2.080358e-3

ActSig1_0 = 7.76e-8

InhSig1_0 = 1.12e-7

CatSig1_0 = 1.2e-4

vSig1_0 = 1.36e-3

AgSig1_0 = 0 }

{7 1+2+34 }

{ oscs_path="7 1+2+34 4oscs\"

Act10 = 7.7893979e-8

Inh10 = 1.1474412e-4

Cat10 = 1.8081622e-5

v10 = 2.080358e-3

Act20 = 7.8181185e-8

Inh20 = 1.5852019e-4

Cat20 = 7.4330636e-5

v20 = 2.1952973e-3

Act30 = 7.7724798e-8

Inh30 = 2.6997116e-4

Cat30 = 5.5233108e-4

v30 = 2.0169811e-3

Act40 = 7.5344063e-8

Inh40 = 2.4148942e-4

Cat40 = 5.9849645e-4

v40 = 1.9673747e-3

ActSig10 = 7.76e-8

InhSig10 = 1.12e-7

CatSig10 = 1.2e-4

vSig10 = 1.36e-3

AgSig10 = 0 }

{8 41+2+3 }

{ oscs_path="8 41+2+3 4oscs\"

Act10 = 7.5344063e-8

Inh10 = 2.4148942e-4

Cat10 = 5.9849645e-4

v10 = 1.9673747e-3

Act20 = 7.7893979e-8

Inh20 = 1.1474412e-4

Cat20 = 1.8081622e-5

v20 = 2.080358e-3

Act30 = 7.8181185e-8

Inh30 = 1.5852019e-4

Cat30 = 7.4330636e-5

v30 = 2.1952973e-3

Act40 = 7.7724798e-8

Inh40 = 2.6997116e-4

Cat40 = 5.5233108e-4

v40 = 2.0169811e-3

ActSig1_0 = 7.76e-8

InhSig1_0 = 1.12e-7

CatSig1_0 = 1.2e-4

vSig1_0 = 1.36e-3

AgSig1_0 = 0 }

{9 1+24+3 }

{ oscs_path="9 1+24+3 4oscs\"

Act10 = 8.71940628529433E-8

Inh10 = 3.52054438378412E-5

Cat10 = 5.55917924221541E-5

v10 = 0.00171665235120331

Act20 = 8.53240278166583E-8

Inh20 = 3.81635426062748E-5

Cat20 = 5.86274347304743E-5

v20 = 0.00171160971066864

Act30 = 1.7741879460252E-7

Inh30 = 8.80759497111896E-6

Cat30 = 2.45614315231313E-5

v30 = 0.00158062459170466

Act40 = 7.88233428974621E-8

Inh40 = 0.000158145568890733

Cat40 = 0.000327153822077095

v40 = 0.00172527021477542

ActSig1_0 = 7.76e-8

InhSig1_0 = 1.12e-7

CatSig1_0 = 1.2e-4

vSig1_0 = 1.36e-3

AgSig1_0 = 0 }

{10 13+2+4}

{ oscs_path="10 13+2+4 4oscs\"

Act10 = 7.5344063e-8

Inh10 = 2.4148942e-4

Cat10 = 5.9849645e-4

v10 = 1.9673747e-3

Act20 = 7.7893979e-8

Inh20 = 1.1474412e-4

Cat20 = 1.8081622e-5

v20 = 2.080358e-3

Act30 = 7.5344063e-8

Inh30 = 2.4148942e-4

Cat30 = 5.9849645e-4

v30 = 1.9673747e-3

Act40 = 7.8181185e-8

Inh40 = 1.5852019e-4

Cat40 = 7.4330636e-5

v40 = 2.1952973e-3

ActSig1_0 = 7.76e-8

InhSig1_0 = 1.12e-7

CatSig1_0 = 1.2e-4

vSig1_0 = 1.36e-3

AgSig1_0 = 0 }

{11 12+4+3 }

{ oscs_path="11 12+4+3 4 oscs\"

Act10 = 7.7724798e-8

Inh10 = 2.6997116e-4

Cat10 = 5.5233108e-4

v10 = 2.0169811e-3

Act20 = 7.5344063e-8

Inh20 = 2.4148942e-4

Cat20 = 5.9849645e-4

v20 = 1.9673747e-3

Act30 = 7.8181185e-8

Inh30 = 1.5852019e-4

Cat30 = 7.4330636e-5

v30 = 2.1952973e-3

Act40 = 7.7893979e-8

Inh40 = 1.1474412e-4

Cat40 = 1.8081622e-5

v40 = 2.080358e-3

ActSig1_0 = 7.76e-8

InhSig1_0 = 1.12e-7

CatSig1_0 = 1.2e-4

vSig1_0 = 1.36e-3

AgSig1_0 = 0 }

{12 23+1+4 }

{ oscs_path="12 23+1+4 4 oscs\"

Act10 = 7.7893979e-8

Inh10 = 1.1474412e-4

Cat10 = 1.8081622e-5

v10 = 2.080358e-3

Act20 = 7.7724798e-8

Inh20 = 2.6997116e-4

Cat20 = 5.5233108e-4

v20 = 2.0169811e-3

Act30 = 7.5344063e-8

Inh30 = 2.4148942e-4

Cat30 = 5.9849645e-4

v30 = 1.9673747e-3

Act40 = 7.8181185e-8

Inh40 = 1.5852019e-4

Cat40 = 7.4330636e-5

v40 = 2.1952973e-3

ActSig1_0 = 7.76e-8

InhSig1_0 = 1.12e-7

CatSig1_0 = 1.2e-4

vSig1_0 = 1.36e-3

AgSig1_0 = 0 }

{13 34+2+1 }

{ oscs_path="13 34+2+1 4 oscs\"

Act10 = 7.8181185e-8

Inh10 = 1.5852019e-4

Cat10 = 7.4330636e-5

v10 = 2.1952973e-3

Act20 = 7.7893979e-8

Inh20 = 1.1474412e-4

Cat20 = 1.8081622e-5

v20 = 2.080358e-3

Act30 = 7.7724798e-8

Inh30 = 2.6997116e-4

Cat30 = 5.5233108e-4

v30 = 2.0169811e-3

Act40 = 7.5344063e-8

Inh40 = 2.4148942e-4

Cat40 = 5.9849645e-4

v40 = 1.9673747e-3

ActSig1_0 = 7.76e-8

InhSig1_0 = 1.12e-7

CatSig1_0 = 1.2e-4

vSig1_0 = 1.36e-3

AgSig1_0 = 0 }

{14 41+3+2 T=160.5 s }

{ oscs_path="14 41+3+2 4 oscs\"

Act10 = 7.7724798e-8

Inh10 = 2.6997116e-4

Cat10 = 5.5233108e-4

v10 = 2.0169811e-3

Act20 = 7.8181185e-8

Inh20 = 1.5852019e-4

Cat20 = 7.4330636e-5

v20 = 2.1952973e-3

Act30 = 7.7893979e-8

Inh30 = 1.1474412e-4

Cat30 = 1.8081622e-5

v30 = 2.080358e-3

Act40 = 7.5344063e-8

Inh40 = 2.4148942e-4

Cat40 = 5.9849645e-4

v40 = 1.9673747e-3

ActSig1_0 = 7.76e-8

InhSig1_0 = 1.12e-7

CatSig1_0 = 1.2e-4

vSig1_0 = 1.36e-3

AgSig1_0 = 0 }

{15 24+1+3 }

{ oscs_path="15 24+1+3 4oscs\"

Act10 = 7.8181185e-8

Inh10 = 1.5852019e-4

Cat10 = 7.4330636e-5

v10 = 2.1952973e-3

Act20 = 7.7893979e-8

Inh20 = 1.1474412e-4

Cat20 = 1.8081622e-5

v20 = 2.080358e-3

Act30 = 7.5344063e-8

Inh30 = 2.4148942e-4

Cat30 = 5.9849645e-4

v30 = 1.9673747e-3

Act40 = 7.7893979e-8

Inh40 = 1.1474412e-4

Cat40 = 1.8081622e-5

v40 = 2.080358e-3

ActSig1_0 = 7.76e-8

InhSig1_0 = 1.12e-7

CatSig1_0 = 1.2e-4

vSig1_0 = 1.36e-3

AgSig1_0 = 0 }

{16 13+4+2 }

{ oscs_path="16 13+4+2 4oscs\"

Act10 = 7.5344063e-8

Inh10 = 2.4148942e-4

Cat10 = 5.9849645e-4

v10 = 1.9673747e-3

Act20 = 7.8181185e-8

Inh20 = 1.5852019e-4

Cat20 = 7.4330636e-5

v20 = 2.1952973e-3

Act30 = 7.5344063e-8

Inh30 = 2.4148942e-4

Cat30 = 5.9849645e-4

v30 = 1.9673747e-3

Act40 = 7.7893979e-8

Inh40 = 1.1474412e-4

Cat40 = 1.8081622e-5

v40 = 2.080358e-3

ActSig1_0 = 7.76e-8

InhSig1_0 = 1.12e-7

CatSig1_0 = 1.2e-4

vSig1_0 = 1.36e-3

AgSig1_0 = 0 }

{17 123+4 }

{ oscs_path="17 123+4 4oscs\"

Act10 = 7.1e-7

Inh10 =1e-7

Cat10 = 3.29342E-5

v10 = 1e-7

Act20 = 7.1e-7

Inh20 =1e-7

Cat20 = 3.29342E-5

v20 = 1e-7

Act30 = 7.1e-7

Inh30 =1e-7

Cat30 = 3.29342E-5

v30 = 1e-7

Act40 = 7.76e-8

Inh40 = 1.12e-7

Cat40 = 1.2e-4

v40 = 1.36e-3

ActSig1_0 = 7.76e-8

InhSig1_0 = 1.12e-7

CatSig1_0 = 1.2e-4

vSig1_0 = 1.36e-3

AgSig1_0 = 0 }

{18 124+3 }

{ oscs_path="18 124+3 4oscs\"

Act10 = 7.1e-7

Inh10 =1e-7

Cat10 = 3.29342E-5

v10 = 1e-7

Act20 = 7.1e-7

Inh20 =1e-7

Cat20 = 3.29342E-5

v20 = 1e-7

Act30 = 7.76e-8

Inh30 = 1.12e-7

Cat30 = 1.2e-4

v30 = 1.36e-3

Act40 = 7.1e-7

Inh40 =1e-7

Cat40 = 3.29342E-5

v40 = 1e-7

ActSig1_0 = 7.76e-8

InhSig1_0 = 1.12e-7

CatSig1_0 = 1.2e-4

vSig1_0 = 1.36e-3

AgSig1_0 = 0 }

{19 134+2 }

{ oscs_path="19 134+2 4oscs\"

Act10 = 7.1e-7

Inh10 =1e-7

Cat10 = 3.29342E-5

v10 = 1e-7

Act20 = 7.76e-8

Inh20 = 1.12e-7

Cat20 = 1.2e-4

v20 = 1.36e-3

Act30 = 7.1e-7

Inh30 =1e-7

Cat30 = 3.29342E-5

v30 = 1e-7

Act40 = 7.1e-7

Inh40 =1e-7

Cat40 = 3.29342E-5

v40 = 1e-7

ActSig1_0 = 7.76e-8

InhSig1_0 = 1.12e-7

CatSig1_0 = 1.2e-4

vSig1_0 = 1.36e-3

AgSig1_0 = 0 }

{20 234+1 }

{ oscs_path="20 234+1 4oscs\"

Act10 = 7.76e-8

Inh10 = 1.12e-7

Cat10 = 1.2e-4

v10 = 1.36e-3

Act20 = 7.1e-7

Inh20 =1e-7

Cat20 = 3.29342E-5

v20 = 1e-7

Act30 = 7.1e-7

Inh30 =1e-7

Cat30 = 3.29342E-5

v30 = 1e-7

Act40 = 7.1e-7

Inh40 =1e-7

Cat40 = 3.29342E-5

v40 = 1e-7

ActSig1_0 = 7.76e-8

InhSig1_0 = 1.12e-7

CatSig1_0 = 1.2e-4

vSig1_0 = 1.36e-3

AgSig1_0 = 0 }

f_act1 = - k11*act1*inh1 + k21*inh1 - 2*k3*act1^2 + k41*act1*{->}(cF - cat1)/(cF - cat1 + cmin) - k0*act1

f_inh1 = -k11*act1*inh1 - k21*inh1 + k9p*v1*cat1 - k0*(inh1) + Cinh*bs4 + Cinh*bs2 + Cinh*bs3

f_cat1 = 2*k41*act1*(cF - cat1)/(cF - cat1 + cmin) - k9p*v1*cat1 - k10*cat1

f_v1 = 2*k11*act1*inh1 + k21*inh1 + k3*act1^2 - k9p*v1*cat1 - k1_3*v1 - k0*v1

f_act2 = - k12*act2*inh2 + k22*inh2 - 2*k3*act2^2 + k42*act2*(cF - cat2)/(cF - cat2 + cmin) - k0*act2

f_inh2 = -1*k12*act2*inh2 - k22*inh2 + k9p*v2*cat2 - k0*(inh2) + Cinh*bs1 + Cinh*bs3 + Cinh*bs4

f_cat2 = 2*k42*act2*(cF - cat2)/(cF - cat2 + cmin) - k9p*v2*cat2 - k10*cat2

f_v2 = 2*k12*act2*inh2 + k22*inh2 + k3*act2^2 - k9p*v2*cat2 - k1_3*v2 - k0*v2

f_act3 = -k13*act3*inh3 + k23*inh3 - 2*k3*act3^2 + k43*act3*(cF - cat3)/(cF - cat3 + cmin) - k0*act3

f_inh3 = -1*k13*act3*inh3 - k23*inh3 + k9p*v3*cat3 - k0*(inh3) + Cinh*bs2 + Cinh*bs1 + Cinh*bs4

f_cat3 = 2*k43*act3*(cF - cat3)/(cF - cat3 + cmin) - k9p*v3*cat3 - k10*cat3

f_v3 = 2*k13*act3*inh3 + k23*inh3 + k3*act3^2 - k9p*v3*cat3 - k1_3*v3 - k0*v3

f_act4 = -k14*act4*inh4 + k24*inh4 - 2*k3*act4^2 + k44*act4*(cF - cat4)/(cF - cat4 + cmin) - k0*act4

f_inh4 = -1*k14*act4*inh4 - k24*inh4 + k9p*v4*cat4 - k0*(inh4) + Cinh*bs3 + Cinh*bs1 + Cinh*bs2

f_cat4 = 2*k44*act4*(cF - cat4)/(cF - cat4 + cmin) - k9p*v4*cat4 - k10*cat4

f_v4 = 2*k14*act4*inh4 + k24*inh4 + k3*act4^2 - k9p*v4*cat4 - k1_3*v4 - k0*v4

f_actSig1 = -k15*actSig1*inhSig1 + k25*inhSig1 - 2*k3*actSig1^2 + k45*actSig1*(cF - catSig1)/(cF - catSig1 + cmin) - k0*actSig1

f_inhSig1 = -k15*actSig1*inhSig1 - k25*inhSig1 + k9p*vSig1*catSig1 - k0*(inhSig1-Inh001) - kdiff*inhSig1*AgSig1

f_catSig1 = 2*k45*actSig1*(cF - catSig1)/(cF - catSig1 + cmin) - k9p*vSig1*catSig1 - k10*catSig1

f_vSig1 = 2*k15*actSig1*inhSig1 + k25*inhSig1 + k3*actSig1^2 - k9p*vSig1*catSig1 - k1_3*vSig1 - k0*vSig1

f_AgSig1 = Cact*(bsSig11 + bsSig12 + bsSig13 + bsSig14) - kdiff*inhSig1*AgSig1

f_actSig2 = -k15*actSig2*inhSig2 + k25*inhSig2 - 2*k3*actSig2^2 + k45*actSig2*(cF - catSig2)/(cF - catSig2 + cmin) - k0*actSig2

f_inhSig2 = -k15*actSig2*inhSig2 - k25*inhSig2 + k9p*vSig2*catSig2 - k0*(inhSig2-Inh002) - kdiff*inhSig2*AgSig2 + CinhSig*bsSig25

f_catSig2 = 2*k45*actSig2*(cF - catSig2)/(cF - catSig2 + cmin) - k9p*vSig2*catSig2 - k10*catSig2

f_vSig2 = 2*k15*actSig2*inhSig2 + k25*inhSig2 + k3*actSig2^2 - k9p*vSig2*catSig2 - k1_3*vSig2 - k0*vSig2

f_AgSig2 = Cact*(bsSig21 + bsSig22 + bsSig23 + bsSig24) - kdiff*inhSig2*AgSig2

f_actSig3 = -k15*actSig3*inhSig3 + k25*inhSig3 - 2*k3*actSig3^2 + k45*actSig3*(cF - catSig3)/(cF - catSig3 + cmin) - k0*actSig3

f_inhSig3 = -k15*actSig3*inhSig3 - k25*inhSig3 + k9p*vSig3*catSig3 - k0*(inhSig3-Inh003) - kdiff*inhSig3*AgSig3 + CinhSig*bsSig35 + CinhSig*bsSig36

f_catSig3 = 2*k45*actSig3*(cF - catSig3)/(cF - catSig3 + cmin) - k9p*vSig3*catSig3 - k10*catSig3

f_vSig3 = 2*k15*actSig3*inhSig3 + k25*inhSig3 + k3*actSig3^2 - k9p*vSig3*catSig3 - k1_3*vSig3 - k0*vSig3

f_AgSig3 = Cact*(bsSig31 + bsSig32 + bsSig33 + bsSig34) - kdiff*inhSig3*AgSig3

f_actSig4 = -k15*actSig4*inhSig4 + k25*inhSig4 - 2*k3*actSig4^2 + k45*actSig4*(cF - catSig4)/(cF - catSig4 + cmin) - k0*actSig4

f_inhSig4 = -k15*actSig4*inhSig4 - k25*inhSig4 + k9p*vSig4*catSig4 - k0*(inhSig4-Inh004) - kdiff*inhSig4*AgSig4 + CinhSig*bsSig45 + CinhSig*bsSig46 + CinhSig*bsSig47

f_catSig4 = 2*k45*actSig4*(cF - catSig4)/(cF - catSig4 + cmin) - k9p*vSig4*catSig4 - k10*catSig4

f_vSig4 = 2*k15*actSig4*inhSig4 + k25*inhSig4 + k3*actSig4^2 - k9p*vSig4*catSig4 - k1_3*vSig4 - k0*vSig4

f_AgSig4 = Cact*(bsSig41 + bsSig42 + bsSig43 + bsSig44) - kdiff*inhSig4*AgSig4

{timers from reactors to reactors}

f_bss1 =b1*(-bss1^3 + (bsr1+bsr2)*bss1^2 - (bsr1*bsr2)*bss1) + 4*ustep(act1-{->}actspike{<-})*ustep(f_act1) - 0.6*ustep(pd1 - 0.5)

f_pd1 = 0.5*(bss1 - Qd1*pd1)/tau_d

f_bs1 = b2*(-bs1^3 + (bsr1+bsr2)*bs1^2 - (bsr1*bsr2)*bs1) + 1*ustep(pd1 - 0.5) - 0.8*ustep(pr1 - 0.6) - bs1*Qbs1

f_pr1 = 0.6*(bs1 - Qr1*pr1)/delta_t

f_bss2 =b1*(-bss2^3 + (bsr1+bsr2)*bss2^2 - (bsr1*bsr2)*bss2) + 4*ustep(act2-actspike)*ustep(f_act2) - 0.6*ustep(pd2 - 0.5)

f_pd2 = 0.5*(bss2 - Qd2*pd2)/tau_d

f_bs2 = b2*(-bs2^3 + (bsr1+bsr2)*bs2^2 - (bsr1*bsr2)*bs2) + 1*ustep(pd2 - 0.5) - 0.8*ustep(pr2 - 0.6) - bs2*Qbs2

f_pr2 = 0.6*(bs2 - Qr2*pr2)/delta_t

f_bss3 =b1*(-bss3^3 + (bsr1+bsr2)*bss3^2 - (bsr1*bsr2)*bss3) + 4*ustep(act3-actspike)*ustep(f_act3) - 0.6*ustep(pd3 - 0.5)

f_pd3 = 0.5*(bss3 - Qd3*pd3)/tau_d

f_bs3 = b2*(-bs3^3 + (bsr1+bsr2)*bs3^2 - (bsr1*bsr2)*bs3) + 1*ustep(pd3 - 0.5) - 0.8*ustep(pr3 - 0.6) - bs3*Qbs3

f_pr3 = 0.6*(bs3 - Qr3*pr3)/delta_t

f_bss4 =b1*(-bss4^3 + (bsr1+bsr2)*bss4^2 - (bsr1*bsr2)*bss4) + 4*ustep(act4-actspike)*ustep(f_act4) - 0.6*ustep(pd4 - 0.5)

f_pd4 = 0.5*(bss4 - Qd4*pd4)/tau_d

f_bs4 = b2*(-bs4^3 + (bsr1+bsr2)*bs4^2 - (bsr1*bsr2)*bs4) + 1*ustep(pd4 - 0.5) - 0.8*ustep(pr4 - 0.6) - bs4*Qbs4

f_pr4 = 0.6*(bs4 - Qr4*pr4)/delta_t

{1}

f_bssSig11 =b1*(-bssSig11^3 + (bsr1+bsr2)*bssSig11^2 - (bsr1*bsr2)*bssSig11) + 4*ustep(act1-actspike)*ustep(f_act1) - 0.6*ustep(pdSig11 - 0.5)

f_pdSig11 = 0.5*(bssSig11 - QdSig11*pdSig11)/(tau_d_coeff11 * period1)

f_bsSig11 = b2*(-bsSig11^3 + (bsr1+bsr2)*bsSig11^2 - (bsr1*bsr2)*bsSig11) + 1*ustep(pdSig11 - 0.5) - 0.8*ustep(prSig11 - 0.6) - bsSig11*QbsSig11

f_prSig11 = 0.6*(bsSig11 - QrSig11*prSig11)/delta_t

f_bssSig12 =b1*(-bssSig12^3 + (bsr1+bsr2)*bssSig12^2 - (bsr1*bsr2)*bssSig12) + 4*ustep(act2-actspike)*ustep(f_act2) - 0.6*ustep(pdSig12 - 0.5)

f_pdSig12 = 0.5*(bssSig12 - QdSig12*pdSig12)/(tau_d_coeff12 * period2)

f_bsSig12 = b2*(-bsSig12^3 + (bsr1+bsr2)*bsSig12^2 - (bsr1*bsr2)*bsSig12) + 1*ustep(pdSig12 - 0.5) - 0.8*ustep(prSig12 - 0.6) - bsSig12*QbsSig12

f_prSig12 = 0.6*(bsSig12 - QrSig12*prSig12)/delta_t

f_bssSig13 =b1*(-bssSig13^3 + (bsr1+bsr2)*bssSig13^2 - (bsr1*bsr2)*bssSig13) + 4*ustep(act3-actspike)*ustep(f_act3) - 0.6*ustep(pdSig13 - 0.5)

f_pdSig13 = 0.5*(bssSig13 - QdSig13*pdSig13)/(tau_d_coeff13 * period3)

f_bsSig13 = b2*(-bsSig13^3 + (bsr1+bsr2)*bsSig13^2 - (bsr1*bsr2)*bsSig13) + 1*ustep(pdSig13 - 0.5) - 0.8*ustep(prSig13 - 0.6) - bsSig13*QbsSig13

f_prSig13 = 0.6*(bsSig13 - QrSig13*prSig13)/delta_t

f_bssSig14 =b1*(-bssSig14^3 + (bsr1+bsr2)*bssSig14^2 - (bsr1*bsr2)*bssSig14) + 4*ustep(act4-actspike)*ustep(f_act4) - 0.6*ustep(pdSig14 - 0.5)

f_pdSig14 = 0.5*(bssSig14 - QdSig14*pdSig14)/(tau_d_coeff14 * period4)

f_bsSig14 = b2*(-bsSig14^3 + (bsr1+bsr2)*bsSig14^2 - (bsr1*bsr2)*bsSig14) + 1*ustep(pdSig14 - 0.5) - 0.8*ustep(prSig14 - 0.6) - bsSig14*QbsSig14

f_prSig14 = 0.6*(bsSig14 - QrSig14*prSig14)/delta_t

{2}

f_bssSig21 =b1*(-bssSig21^3 + (bsr1+bsr2)*bssSig21^2 - (bsr1*bsr2)*bssSig21) + 4*ustep(act1-actspike)*ustep(f_act1) - 0.6*ustep(pdSig21 - 0.5)

f_pdSig21 = 0.5*(bssSig21 - QdSig21*pdSig21)/(tau_d_coeff21 * period1)

f_bsSig21 = b2*(-bsSig21^3 + (bsr1+bsr2)*bsSig21^2 - (bsr1*bsr2)*bsSig21) + 1*ustep(pdSig21 - 0.5) - 0.8*ustep(prSig21 - 0.6) - bsSig21*QbsSig21

f_prSig21 = 0.6*(bsSig21 - QrSig21*prSig21)/delta_t

f_bssSig22 =b1*(-bssSig22^3 + (bsr1+bsr2)*bssSig22^2 - (bsr1*bsr2)*bssSig22) + 4*ustep(act2-actspike)*ustep(f_act2) - 0.6*ustep(pdSig22 - 0.5)

f_pdSig22 = 0.5*(bssSig22 - QdSig22*pdSig22)/(tau_d_coeff22 * period2)

f_bsSig22 = b2*(-bsSig22^3 + (bsr1+bsr2)*bsSig22^2 - (bsr1*bsr2)*bsSig22) + 1*ustep(pdSig22 - 0.5) - 0.8*ustep(prSig22 - 0.6) - bsSig22*QbsSig22

f_prSig22 = 0.6*(bsSig22 - QrSig22*prSig22)/delta_t

f_bssSig23 =b1*(-bssSig23^3 + (bsr1+bsr2)*bssSig23^2 - (bsr1*bsr2)*bssSig23) + 4*ustep(act3-actspike)*ustep(f_act3) - 0.6*ustep(pdSig23 - 0.5)

f_pdSig23 = 0.5*(bssSig23 - QdSig23*pdSig23)/(tau_d_coeff23 * period3)

f_bsSig23 = b2*(-bsSig23^3 + (bsr1+bsr2)*bsSig23^2 - (bsr1*bsr2)*bsSig23) + 1*ustep(pdSig23 - 0.5) - 0.8*ustep(prSig23 - 0.6) - bsSig23*QbsSig23

f_prSig23 = 0.6*(bsSig23 - QrSig23*prSig23)/delta_t

f_bssSig24 =b1*(-bssSig24^3 + (bsr1+bsr2)*bssSig24^2 - (bsr1*bsr2)*bssSig24) + 4*ustep(act4-actspike)*ustep(f_act4) - 0.6*ustep(pdSig24 - 0.5)

f_pdSig24 = 0.5*(bssSig24 - QdSig24*pdSig24)/(tau_d_coeff24 * period4)

f_bsSig24 = b2*(-bsSig24^3 + (bsr1+bsr2)*bsSig24^2 - (bsr1*bsr2)*bsSig24) + 1*ustep(pdSig24 - 0.5) - 0.8*ustep(prSig24 - 0.6) - bsSig24*QbsSig24

f_prSig24 = 0.6*(bsSig24 - QrSig24*prSig24)/delta_t

f_bssSig25 =b1*(-bssSig25^3 + (bsr1+bsr2)*bssSig25^2 - (bsr1*bsr2)*bssSig25) + 4*ustep(actSig1-actspike)*ustep(f_actSig1) - 0.6*ustep(pdSig25 - 0.5)

f_pdSig25= 0.5*(bssSig25 - QdSig25*pdSig25)/(tau_d_coeff25 * period4)

f_bsSig25 = b2*(-bsSig25^3 + (bsr1+bsr2)*bsSig25^2 - (bsr1*bsr2)*bsSig25) + 1*ustep(pdSig25 - 0.5) - 0.8*ustep(prSig25 - 0.6) - bsSig25*QbsSig25

f_prSig25 = 0.6*(bsSig25 - QrSig25*prSig25)/delta_t

{3}

f_bssSig31 =b1*(-bssSig31^3 + (bsr1+bsr2)*bssSig31^2 - (bsr1*bsr2)*bssSig31) + 4*ustep(act1-actspike)*ustep(f_act1) - 0.6*ustep(pdSig31 - 0.5)

f_pdSig31 = 0.5*(bssSig31 - QdSig31*pdSig31)/(tau_d_coeff31 * period1)

f_bsSig31 = b2*(-bsSig31^3 + (bsr1+bsr2)*bsSig31^2 - (bsr1*bsr2)*bsSig31) + 1*ustep(pdSig31 - 0.5) - 0.8*ustep(prSig31 - 0.6) - bsSig31*QbsSig31

f_prSig31 = 0.6*(bsSig31 - QrSig31*prSig31)/delta_t

f_bssSig32 =b1*(-bssSig32^3 + (bsr1+bsr2)*bssSig32^2 - (bsr1*bsr2)*bssSig32) + 4*ustep(act2-actspike)*ustep(f_act2) - 0.6*ustep(pdSig32 - 0.5)

f_pdSig32 = 0.5*(bssSig32 - QdSig32*pdSig32)/(tau_d_coeff32 * period2)

f_bsSig32 = b2*(-bsSig32^3 + (bsr1+bsr2)*bsSig32^2 - (bsr1*bsr2)*bsSig32) + 1*ustep(pdSig32 - 0.5) - 0.8*ustep(prSig32 - 0.6) - bsSig32*QbsSig32

f_prSig32 = 0.6*(bsSig32 - QrSig32*prSig32)/delta_t

f_bssSig33 =b1*(-bssSig33^3 + (bsr1+bsr2)*bssSig33^2 - (bsr1*bsr2)*bssSig33) + 4*ustep(act3-actspike)*ustep(f_act3) - 0.6*ustep(pdSig33 - 0.5)

f_pdSig33 = 0.5*(bssSig33 - QdSig33*pdSig33)/(tau_d_coeff33 * period3)

f_bsSig33 = b2*(-bsSig33^3 + (bsr1+bsr2)*bsSig33^2 - (bsr1*bsr2)*bsSig33) + 1*ustep(pdSig33 - 0.5) - 0.8*ustep(prSig33 - 0.6) - bsSig33*QbsSig33

f_prSig33 = 0.6*(bsSig33 - QrSig33*prSig33)/delta_t

f_bssSig34 =b1*(-bssSig34^3 + (bsr1+bsr2)*bssSig34^2 - (bsr1*bsr2)*bssSig34) + 4*ustep(act4-actspike)*ustep(f_act4) - 0.6*ustep(pdSig34 - 0.5)

f_pdSig34 = 0.5*(bssSig34 - QdSig34*pdSig34)/(tau_d_coeff34 * period4)

f_bsSig34 = b2*(-bsSig34^3 + (bsr1+bsr2)*bsSig34^2 - (bsr1*bsr2)*bsSig34) + 1*ustep(pdSig34 - 0.5) - 0.8*ustep(prSig34 - 0.6) - bsSig34*QbsSig34

f_prSig34 = 0.6*(bsSig34 - QrSig34*prSig34)/delta_t

f_bssSig35 =b1*(-bssSig35^3 + (bsr1+bsr2)*bssSig35^2 - (bsr1*bsr2)*bssSig35) + 4*ustep(actSig1-actspike)*ustep(f_actSig1) - 0.6*ustep(pdSig35 - 0.5)

f_pdSig35 = 0.5*(bssSig35 - QdSig35*pdSig35)/(tau_d_coeff35 * period4)

f_bsSig35 = b2*(-bsSig35^3 + (bsr1+bsr2)*bsSig35^2 - (bsr1*bsr2)*bsSig35) + 1*ustep(pdSig35 - 0.5) - 0.8*ustep(prSig35 - 0.6) - bsSig35*QbsSig35

f_prSig35 = 0.6*(bsSig35 - QrSig35*prSig35)/delta_t

f_bssSig36 =b1*(-bssSig36^3 + (bsr1+bsr2)*bssSig36^2 - (bsr1*bsr2)*bssSig36) + 4*ustep(actSig2-actspike)*ustep(f_actSig2) - 0.6*ustep(pdSig36 - 0.5)

f_pdSig36 = 0.5*(bssSig36 - QdSig36*pdSig36)/(tau_d_coeff36 * period4)

f_bsSig36 = b2*(-bsSig36^3 + (bsr1+bsr2)*bsSig36^2 - (bsr1*bsr2)*bsSig36) + 1*ustep(pdSig36 - 0.5) - 0.8*ustep(prSig36 - 0.6) - bsSig36*QbsSig36

f_prSig36 = 0.6*(bsSig36 - QrSig36*prSig36)/delta_t

{4}

f_bssSig41 =b1*(-bssSig41^3 + (bsr1+bsr2)*bssSig41^2 - (bsr1*bsr2)*bssSig41) + 4*ustep(act1-actspike)*ustep(f_act1) - 0.6*ustep(pdSig41 - 0.5)

f_pdSig41 = 0.5*(bssSig41 - QdSig41*pdSig41)/(tau_d_coeff41 * period1)

f_bsSig41 = b2*(-bsSig41^3 + (bsr1+bsr2)*bsSig41^2 - (bsr1*bsr2)*bsSig41) + 1*ustep(pdSig41 - 0.5) - 0.8*ustep(prSig41 - 0.6) - bsSig41*QbsSig41

f_prSig41 = 0.6*(bsSig41 - QrSig41*prSig41)/delta_t

f_bssSig42 =b1*(-bssSig42^3 + (bsr1+bsr2)*bssSig42^2 - (bsr1*bsr2)*bssSig42) + 4*ustep(act2-actspike)*ustep(f_act2) - 0.6*ustep(pdSig42 - 0.5)

f_pdSig42 = 0.5*(bssSig42 - QdSig42*pdSig42)/(tau_d_coeff42 * period2)

f_bsSig42 = b2*(-bsSig42^3 + (bsr1+bsr2)*bsSig42^2 - (bsr1*bsr2)*bsSig42) + 1*ustep(pdSig42 - 0.5) - 0.8*ustep(prSig42 - 0.6) - bsSig42*QbsSig42

f_prSig42 = 0.6*(bsSig42 - QrSig42*prSig42)/delta_t

f_bssSig43 =b1*(-bssSig43^3 + (bsr1+bsr2)*bssSig43^2 - (bsr1*bsr2)*bssSig43) + 4*ustep(act3-actspike)*ustep(f_act3) - 0.6*ustep(pdSig43 - 0.5)

f_pdSig43 = 0.5*(bssSig43 - QdSig43*pdSig43)/(tau_d_coeff43 * period3)

f_bsSig43 = b2*(-bsSig43^3 + (bsr1+bsr2)*bsSig43^2 - (bsr1*bsr2)*bsSig43) + 1*ustep(pdSig43 - 0.5) - 0.8*ustep(prSig43 - 0.6) - bsSig43*QbsSig43

f_prSig43 = 0.6*(bsSig43 - QrSig43*prSig43)/delta_t

f_bssSig44 =b1*(-bssSig44^3 + (bsr1+bsr2)*bssSig44^2 - (bsr1*bsr2)*bssSig44) + 4*ustep(act4-actspike)*ustep(f_act4) - 0.6*ustep(pdSig44 - 0.5)

f_pdSig44 = 0.5*(bssSig44 - QdSig44*pdSig44)/(tau_d_coeff44 * period4)

f_bsSig44 = b2*(-bsSig44^3 + (bsr1+bsr2)*bsSig44^2 - (bsr1*bsr2)*bsSig44) + 1*ustep(pdSig44 - 0.5) - 0.8*ustep(prSig44 - 0.6) - bsSig44*QbsSig44

f_prSig44 = 0.6*(bsSig44 - QrSig44*prSig44)/delta_t

f_bssSig45 =b1*(-bssSig45^3 + (bsr1+bsr2)*bssSig45^2 - (bsr1*bsr2)*bssSig45) + 4*ustep(actSig1-actspike)*ustep(f_actSig1) - 0.6*ustep(pdSig45 - 0.5)

f_pdSig45 = 0.5*(bssSig45 - QdSig45*pdSig45)/(tau_d_coeff45 * period4)

f_bsSig45 = b2*(-bsSig45^3 + (bsr1+bsr2)*bsSig45^2 - (bsr1*bsr2)*bsSig45) + 1*ustep(pdSig45 - 0.5) - 0.8*ustep(prSig45 - 0.6) - bsSig45*QbsSig45

f_prSig45 = 0.6*(bsSig45 - QrSig45*prSig45)/delta_t

f_bssSig46 =b1*(-bssSig46^3 + (bsr1+bsr2)*bssSig46^2 - (bsr1*bsr2)*bssSig46) + 4*ustep(actSig2-actspike)*ustep(f_actSig2) - 0.6*ustep(pdSig46 - 0.5)

f_pdSig46 = 0.5*(bssSig46 - QdSig46*pdSig46)/(tau_d_coeff46 * period4)

f_bsSig46 = b2*(-bsSig46^3 + (bsr1+bsr2)*bsSig46^2 - (bsr1*bsr2)*bsSig46) + 1*ustep(pdSig46 - 0.5) - 0.8*ustep(prSig46 - 0.6) - bsSig46*QbsSig46

f_prSig46 = 0.6*(bsSig46 - QrSig46*prSig46)/delta_t

f_bssSig47 =b1*(-bssSig47^3 + (bsr1+bsr2)*bssSig47^2 - (bsr1*bsr2)*bssSig47) + 4*ustep(actSig3-actspike)*ustep(f_actSig3) - 0.6*ustep(pdSig47 - 0.5)

f_pdSig47 = 0.5*(bssSig47 - QdSig47*pdSig47)/(tau_d_coeff47 * period4)

f_bsSig47 = b2*(-bsSig47^3 + (bsr1+bsr2)*bsSig47^2 - (bsr1*bsr2)*bsSig47) + 1*ustep(pdSig47 - 0.5) - 0.8*ustep(prSig47 - 0.6) - bsSig47*QbsSig47

f_prSig47 = 0.6*(bsSig47 - QrSig47*prSig47)/delta_t

INITIAL VALUE

Act1 = Act10

Inh1 = Inh10

Cat1 = Cat10

v1 = v10

Act2 = Act20

Inh2 = Inh20

Cat2 = Cat20

v2 = v20

Act3 = Act30

Inh3 = Inh30

Cat3 = Cat30

v3 = v30

Act4 = Act40

Inh4 = Inh40

Cat4 = Cat40

v4 = v40

ActSig1 = ActSig1_0

InhSig1 = InhSig1_0

CatSig1 = CatSig1_0

vSig1 = vSig1_0

AgSig1 = AgSig1_0

ActSig2 = ActSig1_0

InhSig2 = InhSig1_0

CatSig2 = CatSig1_0

vSig2 = vSig1_0

AgSig2 = AgSig1_0

ActSig3 = ActSig1_0

InhSig3 = InhSig1_0

CatSig3 = CatSig1_0

vSig3 = vSig1_0

AgSig3 = AgSig1_0

ActSig4 = ActSig1_0

InhSig4 = InhSig1_0

CatSig4 = CatSig1_0

vSig4 = vSig1_0

AgSig4 = AgSig1_0

bs1 = 0

pr1= 0

bs2 = 0

pr2= 0

bs3 = 0

pr3= 0

bs4 = 0

pr4= 0

bss1=0

pd1=0

bss2=0

pd2=0

bss3=0

pd3=0

bss4=0

pd4=0

bsSig11 = 0

prSig11= 0

bsSig12 = 0

prSig12= 0

bsSig13 = 0

prSig13= 0

bsSig14 = 0

prSig14= 0

bsSig21 = 0

prSig21= 0

bsSig22 = 0

prSig22= 0

bsSig23 = 0

prSig23= 0

bsSig24 = 0

prSig24= 0

bsSig25 = 0

prSig25= 0

bsSig31 = 0

prSig31= 0

bsSig32 = 0

prSig32= 0

bsSig33 = 0

prSig33= 0

bsSig34 = 0

prSig34= 0

bsSig35 = 0

prSig35= 0

bsSig36 = 0

prSig36= 0

bsSig41 = 0

prSig41= 0

bsSig42 = 0

prSig42= 0

bsSig43 = 0

prSig43= 0

bsSig44 = 0

prSig44= 0

bsSig45 = 0

prSig45= 0

bsSig46 = 0

prSig46= 0

bsSig47 = 0

prSig47= 0

bssSig11=0

pdSig11=0

bssSig12=0

pdSig12=0

bssSig13=0

pdSig13=0

bssSig14=0

pdSig14=0

bssSig21=0

pdSig21=0

bssSig22=0

pdSig22=0

bssSig23=0

pdSig23=0

bssSig24=0

pdSig24=0

bssSig25=0

pdSig25=0

bssSig31=0

pdSig31=0

bssSig32=0

pdSig32=0

bssSig33=0

pdSig33=0

bssSig34=0

pdSig34=0

bssSig35=0

pdSig35=0

bssSig36=0

pdSig36=0

bssSig41=0

pdSig41=0

bssSig42=0

pdSig42=0

bssSig43=0

pdSig43=0

bssSig44=0

pdSig44=0

bssSig45=0

pdSig45=0

bssSig46=0

pdSig46=0

bssSig47=0

pdSig47=0

EQUATIONS

Act1: dt(Act1) = f_act1

Inh1: dt(Inh1) = f_inh1

cat1: dt(cat1) = f_cat1

v1: dt(v1) = f_v1

Act2: dt(Act2) = f_act2

Inh2: dt(Inh2) = f_inh2

cat2: dt(cat2) = f_cat2

v2: dt(v2) = f_v2

Act3: dt(Act3) = f_act3

Inh3: dt(Inh3) = f_inh3

cat3: dt(cat3) = f_cat3

v3: dt(v3) = f_v3

Act4: dt(Act4) = f_act4

Inh4: dt(Inh4) = f_inh4

cat4: dt(cat4) = f_cat4

v4: dt(v4) = f_v4

ActSig1: dt(ActSig1) = f_actSig1

InhSig1: dt(InhSig1) = f_inhSig1

catSig1: dt(catSig1) = f_catSig1

vSig1: dt(vSig1) = f_vSig1

agSig1: dt(agSig1) = f_agSig1

ActSig2: dt(ActSig2) = f_actSig2

InhSig2: dt(InhSig2) = f_inhSig2

catSig2: dt(catSig2) = f_catSig2

vSig2: dt(vSig2) = f_vSig2

agSig2: dt(agSig2) = f_agSig2

ActSig3: dt(ActSig3) = f_actSig3

InhSig3: dt(InhSig3) = f_inhSig3

catSig3: dt(catSig3) = f_catSig3

vSig3: dt(vSig3) = f_vSig3

agSig3: dt(agSig3) = f_agSig3

ActSig4: dt(ActSig4) = f_actSig4

InhSig4: dt(InhSig4) = f_inhSig4

catSig4: dt(catSig4) = f_catSig4

vSig4: dt(vSig4) = f_vSig4

agSig4: dt(agSig4) = f_agSig4

BS1: dt(bs1) = f_bs1

PR1: dt(pr1) = f_pr1

BS2: dt(bs2) = f_bs2

PR2: dt(pr2) = f_pr2

BS3: dt(bs3) = f_bs3

PR3: dt(pr3) = f_pr3

BS4: dt(bs4) = f_bs4

PR4: dt(pr4) = f_pr4

BSSIG11: dt(bsSig11) = f_bsSig11

PRSIG11: dt(prSig11) = f_prSig11

BSSIG12: dt(bsSig12) = f_bsSig12

PRSIG12: dt(prSig12) = f_prSig12

BSSIG13: dt(bsSig13) = f_bsSig13

PRSIG13: dt(prSig13) = f_prSig13

BSSIG14: dt(bsSig14) = f_bsSig14

PRSIG14: dt(prSig14) = f_prSig14

BSSIG21: dt(bsSig21) = f_bsSig21

PRSIG21: dt(prSig21) = f_prSig21

BSSIG22: dt(bsSig22) = f_bsSig22

PRSIG22: dt(prSig22) = f_prSig22

BSSIG23: dt(bsSig23) = f_bsSig23

PRSIG23: dt(prSig23) = f_prSig23

BSSIG24: dt(bsSig24) = f_bsSig24

PRSIG24: dt(prSig24) = f_prSig24

BSSIG25: dt(bsSig25) = f_bsSig25

PRSIG25: dt(prSig25) = f_prSig25

BSSIG31: dt(bsSig31) = f_bsSig31

PRSIG31: dt(prSig31) = f_prSig31

BSSIG32: dt(bsSig32) = f_bsSig32

PRSIG32: dt(prSig32) = f_prSig32

BSSIG33: dt(bsSig33) = f_bsSig33

PRSIG33: dt(prSig33) = f_prSig33

BSSIG34: dt(bsSig34) = f_bsSig34

PRSIG34: dt(prSig34) = f_prSig34

BSSIG35: dt(bsSig35) = f_bsSig35

PRSIG35: dt(prSig35) = f_prSig35

BSSIG36: dt(bsSig36) = f_bsSig36

PRSIG36: dt(prSig36) = f_prSig36

BSSIG41: dt(bsSig41) = f_bsSig41

PRSIG41: dt(prSig41) = f_prSig41

BSSIG42: dt(bsSig42) = f_bsSig42

PRSIG42: dt(prSig42) = f_prSig42

BSSIG43: dt(bsSig43) = f_bsSig43

PRSIG43: dt(prSig43) = f_prSig43

BSSIG44: dt(bsSig44) = f_bsSig44

PRSIG44: dt(prSig44) = f_prSig44

BSSIG45: dt(bsSig45) = f_bsSig45

PRSIG45: dt(prSig45) = f_prSig45

BSSIG46: dt(bsSig46) = f_bsSig46

PRSIG46: dt(prSig46) = f_prSig46

BSSIG47: dt(bsSig47) = f_bsSig47

PRSIG47: dt(prSig47) = f_prSig47

BSS1: dt(bss1) = f_bss1

PD1: dt(pd1) = f_pd1

BSS2: dt(bss2) = f_bss2

PD2: dt(pd2) = f_pd2

BSS3: dt(bss3) = f_bss3

PD3: dt(pd3) = f_pd3

BSS4: dt(bss4) = f_bss4

PD4: dt(pd4) = f_pd4

BSSSIG11: dt(bssSig11) = f_bssSig11

PDSIG11: dt(pdSig11) = f_pdSig11

BSSSIG12: dt(bssSig12) = f_bssSig12

PDSIG12: dt(pdSig12) = f_pdSig12

BSSSIG13: dt(bssSig13) = f_bssSig13

PDSIG13: dt(pdSig13) = f_pdSig13

BSSSIG14: dt(bssSig14) = f_bssSig14

PDSIG14: dt(pdSig14) = f_pdSig14

BSSSIG21: dt(bssSig21) = f_bssSig21

PDSIG21: dt(pdSig21) = f_pdSig21

BSSSIG22: dt(bssSig22) = f_bssSig22

PDSIG22: dt(pdSig22) = f_pdSig22

BSSSIG23: dt(bssSig23) = f_bssSig23

PDSIG23: dt(pdSig23) = f_pdSig23

BSSSIG24: dt(bssSig24) = f_bssSig24

PDSIG24: dt(pdSig24) = f_pdSig24

BSSSIG25: dt(bssSig25) = f_bssSig25

PDSIG25: dt(pdSig25) = f_pdSig25

BSSSIG31: dt(bssSig31) = f_bssSig31

PDSIG31: dt(pdSig31) = f_pdSig31

BSSSIG32: dt(bssSig32) = f_bssSig32

PDSIG32: dt(pdSig32) = f_pdSig32

BSSSIG33: dt(bssSig33) = f_bssSig33

PDSIG33: dt(pdSig33) = f_pdSig33

BSSSIG34: dt(bssSig34) = f_bssSig34

PDSIG34: dt(pdSig34) = f_pdSig34

BSSSIG35: dt(bssSig35) = f_bssSig35

PDSIG35: dt(pdSig35) = f_pdSig35

BSSSIG36: dt(bssSig36) = f_bssSig36

PDSIG36: dt(pdSig36) = f_pdSig36

BSSSIG41: dt(bssSig41) = f_bssSig41

PDSIG41: dt(pdSig41) = f_pdSig41

BSSSIG42: dt(bssSig42) = f_bssSig42

PDSIG42: dt(pdSig42) = f_pdSig42

BSSSIG43: dt(bssSig43) = f_bssSig43

PDSIG43: dt(pdSig43) = f_pdSig43

BSSSIG44: dt(bssSig44) = f_bssSig44

PDSIG44: dt(pdSig44) = f_pdSig44

BSSSIG45: dt(bssSig45) = f_bssSig45

PDSIG45: dt(pdSig45) = f_pdSig45

BSSSIG46: dt(bssSig46) = f_bssSig46

PDSIG46: dt(pdSig46) = f_pdSig46

BSSSIG47: dt(bssSig47) = f_bssSig47

PDSIG47: dt(pdSig47) = f_pdSig47

BOUNDARIES

region 1

start (0) line to (1)

time time_ini to time_end by 0.00000001

MONITORS

for t = 0 by 100 to time_end

PLOTS

for t = 0 by 500 to time_end

history(act1, act2, act3, act4) at (0.5,0.5) export format "#t#r#b#i" file="result\"+oscs_path+"1 act.txt"

history(actSig1) at (0.5,0.5) export format "#t#r#b#i" file="result\"+oscs_path+"1 IP signal set to\5 actSignal.txt"

history(actSig2) at (0.5,0.5) export format "#t#r#b#i" file="result\"+oscs_path+"2 3Cl signal set to\5 actSignal.txt"

history(actSig3) at (0.5,0.5) export format "#t#r#b#i" file="result\"+oscs_path+"3 2Cl signal set to\5 actSignal.txt"

history(actSig4) at (0.5,0.5) export format "#t#r#b#i" file="result\"+oscs_path+"4 1Cl signal set to\5 actSignal.txt"

history(bsSig25,bsSig35,bsSig45) at (0.5,0.5) !export format "#t#r#b#i" file="result\"+oscs_path+"4 1Cl signal set to\10 bs.txt"

END
