# Supplementary material for: A ‘reader’ unit of the chemical computer
Source: R Soc Open Sci. 2018 Jan 10;5(1):171495. doi: 10.1098/rsos.171495 (PMC5792929; doi:10.1098/rsos.171495)
Supplement: S4. The FlexPDE script [file rsos171495supp4.docx]

TITLE

'ODE'

SELECT

ngrid=1

changelim=0.1

ERRLIM= 0.01

COORDINATES

cartesian1

VARIABLES

Act1

Inh1

Cat1

v1

Act2

Inh2

Cat2

v2

Act3

Inh3

Cat3

v3

Act4

Inh4

Cat4

v4

ActSig1

InhSig1

CatSig1

vSig1

AgSig1(threshold = 0.01)

ActSig2

InhSig2

CatSig2

vSig2

AgSig2(threshold = 0.01)

ActSig3

InhSig3

CatSig3

vSig3

AgSig3(threshold = 0.01)

ActSig4

InhSig4

CatSig4

vSig4

AgSig4(threshold = 0.01)

ActSig5

InhSig5

CatSig5

vSig5

AgSig5(threshold = 0.01)

ActSig6

InhSig6

CatSig6

vSig6

AgSig6(threshold = 0.01)

bs1 (threshold = 0.01)

Pr1 (threshold = 0.01)

bs2 (threshold = 0.01)

Pr2 (threshold = 0.01)

bs3 (threshold = 0.01)

Pr3 (threshold = 0.01)

bs4 (threshold = 0.01)

Pr4 (threshold = 0.01)

bss1 (threshold = 0.01)

Pd1 (threshold = 0.01)

bss2 (threshold = 0.01)

Pd2 (threshold = 0.01)

bss3 (threshold = 0.01)

Pd3 (threshold = 0.01)

bss4 (threshold = 0.01)

Pd4 (threshold = 0.01)

bsSig11 (threshold = 0.01)

PrSig11 (threshold = 0.01)

bsSig12 (threshold = 0.01)

PrSig12 (threshold = 0.01)

bsSig21 (threshold = 0.01)

PrSig21 (threshold = 0.01)

bsSig23 (threshold = 0.01)

PrSig23 (threshold = 0.01)

bsSig31 (threshold = 0.01)

PrSig31 (threshold = 0.01)

bsSig34 (threshold = 0.01)

PrSig34 (threshold = 0.01)

bsSig42 (threshold = 0.01)

PrSig42 (threshold = 0.01)

bsSig43 (threshold = 0.01)

PrSig43 (threshold = 0.01)

bsSig52 (threshold = 0.01)

PrSig52 (threshold = 0.01)

bsSig54 (threshold = 0.01)

PrSig54 (threshold = 0.01)

bsSig63 (threshold = 0.01)

PrSig63 (threshold = 0.01)

bsSig64 (threshold = 0.01)

PrSig64 (threshold = 0.01)

bssSig11 (threshold = 0.01)

PdSig11 (threshold = 0.01)

bssSig12 (threshold = 0.01)

PdSig12 (threshold = 0.01)

bssSig21 (threshold = 0.01)

PdSig21 (threshold = 0.01)

bssSig23 (threshold = 0.01)

PdSig23 (threshold = 0.01)

bssSig31 (threshold = 0.01)

PdSig31 (threshold = 0.01)

bssSig34 (threshold = 0.01)

PdSig34 (threshold = 0.01)

bssSig42 (threshold = 0.01)

PdSig42 (threshold = 0.01)

bssSig43 (threshold = 0.01)

PdSig43 (threshold = 0.01)

bssSig52 (threshold = 0.01)

PdSig52 (threshold = 0.01)

bssSig54 (threshold = 0.01)

PdSig54 (threshold = 0.01)

bssSig63 (threshold = 0.01)

PdSig63 (threshold = 0.01)

bssSig64 (threshold = 0.01)

PdSig64 (threshold = 0.01)

DEFINITIONS

h0 = 0.3

heps = 0.00

h1 = h0 + heps

h2 = h0 - heps

h3 = h0 + heps/2

h4 = h0 - heps/2

h5 = h0

A = 0.25

MA = 0.1

CF = 1e-3

k0 = 5e-4

bsr1 = 0.5

bsr2 = 1

b1 = 3

b2 = 3

k11 = 2.00E+06*h1

k12 = 2.00E+06*h2

k13 = 2.00E+06*h3

k14 = 2.00E+06*h4

k15 = 2.00E+06*h5

k21 = 2*h1*h1*A

k22 = 2*h2*h2*A

k23 = 2*h3*h3*A

k24 = 2*h4*h4*A

k25 = 2*h5*h5*A

k3 = 3000

k41 = 42*h1*A

k42 = 42*h2*A

k43 = 42*h3*A

k44 = 42*h4*A

k45 = 42*h5*A

kr = 2E+08

kred = 5E+06

k9p = 20

k10p = 0.05

k10 = k10p*MA

k1_3 = 0.004

cmin =sqrt(3*kr* k10*CF)/kred

time_ini = 0

time_end = 1300

actspike = 1e-4

Cinh = 2e-5

CinhSig = 2e-2

Cact = 2.5e-6

Inh003 = 4e-3

kdiff = 1e8

delta_t = 5

tau_d = 8 !AP = 1; IP = 40, 50; W = 50; Wr = 20, 30; 3Cl = 7 (a-t-a); 2Cl = 8 (a-t-a); - for initial conditions

period1 = 1 !AP = 123, IP = 114.5, W = 99.8, Wr = 112.8, 3Cl = 160.3, 2Cl = 158

period2 = period1

period3 = period1

period4 = period1

tau_d_coeff11 = 0.1

tau_d_coeff12 = 0.1

tau_d_coeff21 = 0.1

tau_d_coeff23 = 0.1

tau_d_coeff31 = 0.1

tau_d_coeff34 = 0.1

tau_d_coeff42 = 0.1

tau_d_coeff43 = 0.1

tau_d_coeff52 = 0.1

tau_d_coeff54 = 0.1

tau_d_coeff63 = 0.1

tau_d_coeff64 = 0.1

Qr1 = IF bs1 > 0.5 THEN 0 ELSE 5*delta_t

Qr2 = IF bs2 > 0.5 THEN 0 ELSE 5*delta_t

Qr3 = IF bs3 > 0.5 THEN 0 ELSE 5*delta_t

Qr4 = IF bs4 > 0.5 THEN 0 ELSE 5*delta_t

Qd1 = IF bss1 > 0.5 THEN 0 ELSE 5*tau_d

Qd2 = IF bss2 > 0.5 THEN 0 ELSE 5*tau_d

Qd3 = IF bss3 > 0.5 THEN 0 ELSE 5*tau_d

Qd4 = IF bss4 > 0.5 THEN 0 ELSE 5*tau_d

Qbs1 = IF bs1 > 0.5 THEN 0 ELSE 0.5

Qbs2 = IF bs2 > 0.5 THEN 0 ELSE 0.5

Qbs3 = IF bs3 > 0.5 THEN 0 ELSE 0.5

Qbs4 = IF bs4 > 0.5 THEN 0 ELSE 0.5

QrSig11 = IF bsSig11 > 0.5 THEN 0 ELSE 5*delta_t

QrSig12 = IF bsSig12 > 0.5 THEN 0 ELSE 5*delta_t

QdSig11 = IF bssSig11 > 0.5 THEN 0 ELSE 5*tau_d_coeff11*period1

QdSig12 = IF bssSig12 > 0.5 THEN 0 ELSE 5*tau_d_coeff12*period2

QbsSig11 = IF bsSig11 > 0.5 THEN 0 ELSE 0.5

QbsSig12 = IF bsSig12 > 0.5 THEN 0 ELSE 0.5

QrSig21 = IF bsSig21 > 0.5 THEN 0 ELSE 5*delta_t

QrSig23 = IF bsSig23 > 0.5 THEN 0 ELSE 5*delta_t

QdSig21 = IF bssSig21 > 0.5 THEN 0 ELSE 5*tau_d_coeff21*period1

QdSig23 = IF bssSig23 > 0.5 THEN 0 ELSE 5*tau_d_coeff23*period3

QbsSig21 = IF bsSig21 > 0.5 THEN 0 ELSE 0.5

QbsSig23 = IF bsSig23 > 0.5 THEN 0 ELSE 0.5

QrSig31 = IF bsSig31 > 0.5 THEN 0 ELSE 5*delta_t

QrSig34 = IF bsSig34 > 0.5 THEN 0 ELSE 5*delta_t

QdSig31 = IF bssSig31 > 0.5 THEN 0 ELSE 5*tau_d_coeff31*period1

QdSig34 = IF bssSig34 > 0.5 THEN 0 ELSE 5*tau_d_coeff34*period4

QbsSig31 = IF bsSig31 > 0.5 THEN 0 ELSE 0.5

QbsSig34 = IF bsSig34 > 0.5 THEN 0 ELSE 0.5

QrSig42 = IF bsSig42 > 0.5 THEN 0 ELSE 5*delta_t

QrSig43 = IF bsSig43 > 0.5 THEN 0 ELSE 5*delta_t

QdSig42 = IF bssSig42 > 0.5 THEN 0 ELSE 5*tau_d_coeff42*period2

QdSig43 = IF bssSig43 > 0.5 THEN 0 ELSE 5*tau_d_coeff43*period3

QbsSig42 = IF bsSig42 > 0.5 THEN 0 ELSE 0.5

QbsSig43 = IF bsSig43 > 0.5 THEN 0 ELSE 0.5

QrSig52 = IF bsSig52 > 0.5 THEN 0 ELSE 5*delta_t

QrSig54 = IF bsSig54 > 0.5 THEN 0 ELSE 5*delta_t

QdSig52 = IF bssSig52 > 0.5 THEN 0 ELSE 5*tau_d_coeff52*period2

QdSig54 = IF bssSig54 > 0.5 THEN 0 ELSE 5*tau_d_coeff54*period3

QbsSig52 = IF bsSig52 > 0.5 THEN 0 ELSE 0.5

QbsSig54 = IF bsSig54 > 0.5 THEN 0 ELSE 0.5

QrSig63 = IF bsSig63 > 0.5 THEN 0 ELSE 5*delta_t

QrSig64 = IF bsSig64 > 0.5 THEN 0 ELSE 5*delta_t

QdSig63 = IF bssSig63 > 0.5 THEN 0 ELSE 5*tau_d_coeff63*period2

QdSig64 = IF bssSig64 > 0.5 THEN 0 ELSE 5*tau_d_coeff64*period3

QbsSig63 = IF bsSig63 > 0.5 THEN 0 ELSE 0.5

QbsSig64 = IF bsSig64 > 0.5 THEN 0 ELSE 0.5

{Initial values}

{1 AP }

{ oscs_path="1 AP 4oscs\"

Act10 = 7.1e-7

Inh10 =1e-7

Cat10 = 3.29342E-5

v10 = 1e-7

Act20 =7.76e-8

Inh20 = 1.12e-7

Cat20 = 1.2e-4

v20 = 1.36e-3

Act30 = 7.1e-7

Inh30 =1e-7

Cat30 = 3.29342E-5

v30 = 1e-7

Act40 = 7.76e-8

Inh40 = 1.12e-7

Cat40 = 1.2e-4

v40 = 1.36e-3

ActSig1_0 = 7.76e-8

InhSig1_0 = 1.12e-7

CatSig1_0 = 1.2e-4

vSig1_0 = 1.36e-3

AgSig1_0 = 0 }

{2 IP -> 1+234 Cinh = 2e-5 tau_d = 8 a-t-a}

{ oscs_path="2 IP 4oscs\"

Act10 = 0.000120394851493317

Inh10 = 2.73192731536462E-7

Cat10 = 0.000302666706771218

v10 = 0.00456601011752023

Act20 = 2.11007022809961E-7

Inh20 = 9.17926951929157E-6

Cat20 = 1.38574910923001E-5

v20 = 0.0045775865331391

Act30 = 3.73131772272833E-7

Inh30 = 6.62729773256394E-6

Cat30 = 1.6877527273958E-5

v30 = 0.00459045122483998

Act40 = 2.43539283655869E-7

Inh40 = 8.34217616499501E-6

Cat40 = 1.45023881912688E-5

v40 = 0.00458774010992285

ActSig1_0 = 7.76e-8

InhSig1_0 = 1.12e-7

CatSig1_0 = 1.2e-4

vSig1_0 = 1.36e-3

AgSig1_0 = 0 }

{2 IP -> 2+ 134 Cinh = 2e-5 tau_d = 8 a-t-a}

{ oscs_path="2 IP 4oscs\"

Act10 = 7.1e-7

Inh10 =1e-7

Cat10 = 3.29342E-5

v10 = 1e-7

Act20 = 7.1e-7

Inh20 =1e-7

Cat20 = 3.29342E-5

v20 = 1e-7

Act30 = 7.1e-7

Inh30 =1e-7

Cat30 = 3.29342E-5

v30 = 1e-7

Act40 = 7.1e-7

Inh40 =1e-7

Cat40 = 3.29342E-5

v40 = 1e-7

ActSig1_0 = 7.76e-8

InhSig1_0 = 1.12e-7

CatSig1_0 = 1.2e-4

vSig1_0 = 1.36e-3

AgSig1_0 = 0 }

{3 W }

{ oscs_path="3 W 4oscs\"

Act10 = 7.71776479613043E-8

Inh10 = 0.000142379639466751

Cat10 = 0.000254305487778619

v10 = 0.00167433504170452

Act20 = 7.85113049354742E-8

Inh20 = 0.000136040354581379

Cat20 = 0.000783185715106246

v20 = 0.00160135135598607

Act30 = 9.97378691809549E-8

Inh30 = 2.19073279666893E-5

Cat30 = 3.36333291303531E-5

v30 = 0.00154833441905765

Act40 = 8.14222138745477E-8

Inh40 = 5.4966577209892E-5

Cat40 = 9.31964326125288E-5

v40 = 0.00166132632155534

ActSig1_0 = 7.76e-8

InhSig1_0 = 1.12e-7

CatSig1_0 = 1.2e-4

vSig1_0 = 1.36e-3

AgSig1_0 = 0 }

{4 Wr }

{ oscs_path="4 Wr 4oscs\"

Act10 = 8.14222138745477E-8

Inh10 = 5.4966577209892E-5

Cat10 = 9.31964326125288E-5

v10 = 0.00166132632155534

Act20 = 9.97378691809549E-8

Inh20 = 2.19073279666893E-5

Cat20 = 3.36333291303531E-5

v20 = 0.00154833441905765

Act30 = 7.85113049354742E-8

Inh30 = 0.000136040354581379

Cat30 = 0.000783185715106246

v30 = 0.00160135135598607

Act40 = 7.71776479613043E-8

Inh40 = 0.000142379639466751

Cat40 = 0.000254305487778619

v40 = 0.00167433504170452

ActSig1_0 = 7.76e-8

InhSig1_0 = 1.12e-7

CatSig1_0 = 1.2e-4

vSig1_0 = 1.36e-3

AgSig1_0 = 0 }

{5 12+3+4 }

{ oscs_path="5 12+3+4 4oscs\"

Act10 = 7.7724798e-8

Inh10 = 2.6997116e-4

Cat10 = 5.5233108e-4

v10 = 2.0169811e-3

Act20 = 7.5344063e-8

Inh20 = 2.4148942e-4

Cat20 = 5.9849645e-4

v20 = 1.9673747e-3

Act30 = 7.7893979e-8

Inh30 = 1.1474412e-4

Cat30 = 1.8081622e-5

v30 = 2.080358e-3

Act40 = 7.8181185e-8

Inh40 = 1.5852019e-4

Cat40 = 7.4330636e-5

v40 = 2.1952973e-3

ActSig1_0 = 7.76e-8

InhSig1_0 = 1.12e-7

CatSig1_0 = 1.2e-4

vSig1_0 = 1.36e-3

AgSig1_0 = 0 }

{6 1+23+4 }

{ oscs_path="6 1+23+4 4oscs\"

Act10 = 7.8181185e-8

Inh10 = 1.5852019e-4

Cat10 = 7.4330636e-5

v10 = 2.1952973e-3

Act20 = 7.7724798e-8

Inh20 = 2.6997116e-4

Cat20 = 5.5233108e-4

v20 = 2.0169811e-3

Act30 = 7.5344063e-8

Inh30 = 2.4148942e-4

Cat30 = 5.9849645e-4

v30 = 1.9673747e-3

Act40 = 7.7893979e-8

Inh40 = 1.1474412e-4

Cat40 = 1.8081622e-5

v40 = 2.080358e-3

ActSig1_0 = 7.76e-8

InhSig1_0 = 1.12e-7

CatSig1_0 = 1.2e-4

vSig1_0 = 1.36e-3

AgSig1_0 = 0 }

{7 1+2+34 }

{ oscs_path="7 1+2+34 4oscs\"

Act10 = 7.7893979e-8

Inh10 = 1.1474412e-4

Cat10 = 1.8081622e-5

v10 = 2.080358e-3

Act20 = 7.8181185e-8

Inh20 = 1.5852019e-4

Cat20 = 7.4330636e-5

v20 = 2.1952973e-3

Act30 = 7.7724798e-8

Inh30 = 2.6997116e-4

Cat30 = 5.5233108e-4

v30 = 2.0169811e-3

Act40 = 7.5344063e-8

Inh40 = 2.4148942e-4

Cat40 = 5.9849645e-4

v40 = 1.9673747e-3

ActSig1_0 = 7.76e-8

InhSig1_0 = 1.12e-7

CatSig1_0 = 1.2e-4

vSig1_0 = 1.36e-3

AgSig1_0 = 0 }

{8 41+2+3 }

{ oscs_path="8 41+2+3 4oscs\"

Act10 = 7.5344063e-8

Inh10 = 2.4148942e-4

Cat10 = 5.9849645e-4

v10 = 1.9673747e-3

Act20 = 7.7893979e-8

Inh20 = 1.1474412e-4

Cat20 = 1.8081622e-5

v20 = 2.080358e-3

Act30 = 7.8181185e-8

Inh30 = 1.5852019e-4

Cat30 = 7.4330636e-5

v30 = 2.1952973e-3

Act40 = 7.7724798e-8

Inh40 = 2.6997116e-4

Cat40 = 5.5233108e-4

v40 = 2.0169811e-3

ActSig1_0 = 7.76e-8

InhSig1_0 = 1.12e-7

CatSig1_0 = 1.2e-4

vSig1_0 = 1.36e-3

AgSig1_0 = 0 }

{9 1+24+3 }

{ oscs_path="9 1+24+3 4oscs\"

Act10 = 7.88233428974621E-8

Inh10 = 0.000158145568890733

Cat10 = 0.000327153822077095

v10 = 0.00172527021477542

Act20 = 8.71940628529433E-8

Inh20 = 3.52054438378412E-5

Cat20 = 5.55917924221541E-5

v20 = 0.00171665235120331

Act30 = 1.7741879460252E-7

Inh30 = 8.80759497111896E-6

Cat30 = 2.45614315231313E-5

v30 = 0.00158062459170466

Act40 = 8.53240278166583E-8

Inh40 = 3.81635426062748E-5

Cat40 = 5.86274347304743E-5

v40 = 0.00171160971066864

ActSig1_0 = 7.76e-8

InhSig1_0 = 1.12e-7

CatSig1_0 = 1.2e-4

vSig1_0 = 1.36e-3

AgSig1_0 = 0 }

{10 13+2+4}

oscs_path="10 13+2+4 4oscs\"

Act10 = 7.5344063e-8

Inh10 = 2.4148942e-4

Cat10 = 5.9849645e-4

v10 = 1.9673747e-3

Act20 = 7.7893979e-8

Inh20 = 1.1474412e-4

Cat20 = 1.8081622e-5

v20 = 2.080358e-3

Act30 = 7.5344063e-8

Inh30 = 2.4148942e-4

Cat30 = 5.9849645e-4

v30 = 1.9673747e-3

Act40 = 7.8181185e-8

Inh40 = 1.5852019e-4

Cat40 = 7.4330636e-5

v40 = 2.1952973e-3

ActSig1_0 = 7.76e-8

InhSig1_0 = 1.12e-7

CatSig1_0 = 1.2e-4

vSig1_0 = 1.36e-3

AgSig1_0 = 0

{11 12+4+3 }

{ oscs_path="11 12+4+3 4 oscs\"

Act10 = 7.7724798e-8

Inh10 = 2.6997116e-4

Cat10 = 5.5233108e-4

v10 = 2.0169811e-3

Act20 = 7.5344063e-8

Inh20 = 2.4148942e-4

Cat20 = 5.9849645e-4

v20 = 1.9673747e-3

Act30 = 7.8181185e-8

Inh30 = 1.5852019e-4

Cat30 = 7.4330636e-5

v30 = 2.1952973e-3

Act40 = 7.7893979e-8

Inh40 = 1.1474412e-4

Cat40 = 1.8081622e-5

v40 = 2.080358e-3

ActSig1_0 = 7.76e-8

InhSig1_0 = 1.12e-7

CatSig1_0 = 1.2e-4

vSig1_0 = 1.36e-3

AgSig1_0 = 0 }

{12 23+1+4 }

{ oscs_path="12 23+1+4 4 oscs\"

Act10 = 7.7893979e-8

Inh10 = 1.1474412e-4

Cat10 = 1.8081622e-5

v10 = 2.080358e-3

Act20 = 7.7724798e-8

Inh20 = 2.6997116e-4

Cat20 = 5.5233108e-4

v20 = 2.0169811e-3

Act30 = 7.5344063e-8

Inh30 = 2.4148942e-4

Cat30 = 5.9849645e-4

v30 = 1.9673747e-3

Act40 = 7.8181185e-8

Inh40 = 1.5852019e-4

Cat40 = 7.4330636e-5

v40 = 2.1952973e-3

ActSig1_0 = 7.76e-8

InhSig1_0 = 1.12e-7

CatSig1_0 = 1.2e-4

vSig1_0 = 1.36e-3

AgSig1_0 = 0 }

{13 34+2+1 }

{ oscs_path="13 34+2+1 4 oscs\"

Act10 = 7.8181185e-8

Inh10 = 1.5852019e-4

Cat10 = 7.4330636e-5

v10 = 2.1952973e-3

Act20 = 7.7893979e-8

Inh20 = 1.1474412e-4

Cat20 = 1.8081622e-5

v20 = 2.080358e-3

Act30 = 7.7724798e-8

Inh30 = 2.6997116e-4

Cat30 = 5.5233108e-4

v30 = 2.0169811e-3

Act40 = 7.5344063e-8

Inh40 = 2.4148942e-4

Cat40 = 5.9849645e-4

v40 = 1.9673747e-3

ActSig1_0 = 7.76e-8

InhSig1_0 = 1.12e-7

CatSig1_0 = 1.2e-4

vSig1_0 = 1.36e-3

AgSig1_0 = 0 }

{14 41+3+2 T=160.5 s }

{ oscs_path="14 41+3+2 4 oscs\"

Act10 = 7.7724798e-8

Inh10 = 2.6997116e-4

Cat10 = 5.5233108e-4

v10 = 2.0169811e-3

Act20 = 7.8181185e-8

Inh20 = 1.5852019e-4

Cat20 = 7.4330636e-5

v20 = 2.1952973e-3

Act30 = 7.7893979e-8

Inh30 = 1.1474412e-4

Cat30 = 1.8081622e-5

v30 = 2.080358e-3

Act40 = 7.5344063e-8

Inh40 = 2.4148942e-4

Cat40 = 5.9849645e-4

v40 = 1.9673747e-3

ActSig1_0 = 7.76e-8

InhSig1_0 = 1.12e-7

CatSig1_0 = 1.2e-4

vSig1_0 = 1.36e-3

AgSig1_0 = 0 }

{15 24+1+3 }

{ oscs_path="15 24+1+3 4oscs\"

Act10 = 7.8181185e-8

Inh10 = 1.5852019e-4

Cat10 = 7.4330636e-5

v10 = 2.1952973e-3

Act20 = 7.7893979e-8

Inh20 = 1.1474412e-4

Cat20 = 1.8081622e-5

v20 = 2.080358e-3

Act30 = 7.5344063e-8

Inh30 = 2.4148942e-4

Cat30 = 5.9849645e-4

v30 = 1.9673747e-3

Act40 = 7.7893979e-8

Inh40 = 1.1474412e-4

Cat40 = 1.8081622e-5

v40 = 2.080358e-3

ActSig1_0 = 7.76e-8

InhSig1_0 = 1.12e-7

CatSig1_0 = 1.2e-4

vSig1_0 = 1.36e-3

AgSig1_0 = 0 }

{16 13+4+2 }

{ oscs_path="16 13+4+2 4oscs\"

Act10 = 7.5344063e-8

Inh10 = 2.4148942e-4

Cat10 = 5.9849645e-4

v10 = 1.9673747e-3

Act20 = 7.8181185e-8

Inh20 = 1.5852019e-4

Cat20 = 7.4330636e-5

v20 = 2.1952973e-3

Act30 = 7.5344063e-8

Inh30 = 2.4148942e-4

Cat30 = 5.9849645e-4

v30 = 1.9673747e-3

Act40 = 7.7893979e-8

Inh40 = 1.1474412e-4

Cat40 = 1.8081622e-5

v40 = 2.080358e-3

ActSig1_0 = 7.76e-8

InhSig1_0 = 1.12e-7

CatSig1_0 = 1.2e-4

vSig1_0 = 1.36e-3

AgSig1_0 = 0 }

{17 123+4 }

{ oscs_path="17 123+4 4oscs\"

Act10 = 7.1e-7

Inh10 =1e-7

Cat10 = 3.29342E-5

v10 = 1e-7

Act20 = 7.1e-7

Inh20 =1e-7

Cat20 = 3.29342E-5

v20 = 1e-7

Act30 = 7.1e-7

Inh30 =1e-7

Cat30 = 3.29342E-5

v30 = 1e-7

Act40 = 7.76e-8

Inh40 = 1.12e-7

Cat40 = 1.2e-4

v40 = 1.36e-3

ActSig1_0 = 7.76e-8

InhSig1_0 = 1.12e-7

CatSig1_0 = 1.2e-4

vSig1_0 = 1.36e-3

AgSig1_0 = 0 }

{18 124+3 }

{ oscs_path="18 124+3 4oscs\"

Act10 = 7.1e-7

Inh10 =1e-7

Cat10 = 3.29342E-5

v10 = 1e-7

Act20 = 7.1e-7

Inh20 =1e-7

Cat20 = 3.29342E-5

v20 = 1e-7

Act30 = 7.76e-8

Inh30 = 1.12e-7

Cat30 = 1.2e-4

v30 = 1.36e-3

Act40 = 7.1e-7

Inh40 =1e-7

Cat40 = 3.29342E-5

v40 = 1e-7

ActSig1_0 = 7.76e-8

InhSig1_0 = 1.12e-7

CatSig1_0 = 1.2e-4

vSig1_0 = 1.36e-3

AgSig1_0 = 0 }

{19 134+2 }

{ oscs_path="19 134+2 4oscs\"

Act10 = 7.1e-7

Inh10 =1e-7

Cat10 = 3.29342E-5

v10 = 1e-7

Act20 = 7.76e-8

Inh20 = 1.12e-7

Cat20 = 1.2e-4

v20 = 1.36e-3

Act30 = 7.1e-7

Inh30 =1e-7

Cat30 = 3.29342E-5

v30 = 1e-7

Act40 = 7.1e-7

Inh40 =1e-7

Cat40 = 3.29342E-5

v40 = 1e-7

ActSig1_0 = 7.76e-8

InhSig1_0 = 1.12e-7

CatSig1_0 = 1.2e-4

vSig1_0 = 1.36e-3

AgSig1_0 = 0 }

{20 234+1 }

{ oscs_path="20 234+1 4oscs\"

Act10 = 7.76e-8

Inh10 = 1.12e-7

Cat10 = 1.2e-4

v10 = 1.36e-3

Act20 = 7.1e-7

Inh20 =1e-7

Cat20 = 3.29342E-5

v20 = 1e-7

Act30 = 7.1e-7

Inh30 =1e-7

Cat30 = 3.29342E-5

v30 = 1e-7

Act40 = 7.1e-7

Inh40 =1e-7

Cat40 = 3.29342E-5

v40 = 1e-7

ActSig1_0 = 7.76e-8

InhSig1_0 = 1.12e-7

CatSig1_0 = 1.2e-4

vSig1_0 = 1.36e-3

AgSig1_0 = 0 }

f_act1 = - k11*act1*inh1 + k21*inh1 - 2*k3*act1^2 + k41*act1*{->}(cF - cat1)/(cF - cat1 + cmin) - k0*act1

f_inh1 = -k11*act1*inh1 - k21*inh1 + k9p*v1*cat1 - k0*(inh1) + Cinh*bs4 + Cinh*bs2 + Cinh*bs3

f_cat1 = 2*k41*act1*(cF - cat1)/(cF - cat1 + cmin) - k9p*v1*cat1 - k10*cat1

f_v1 = 2*k11*act1*inh1 + k21*inh1 + k3*act1^2 - k9p*v1*cat1 - k1_3*v1 - k0*v1

f_act2 = - k12*act2*inh2 + k22*inh2 - 2*k3*act2^2 + k42*act2*(cF - cat2)/(cF - cat2 + cmin) - k0*act2

f_inh2 = -1*k12*act2*inh2 - k22*inh2 + k9p*v2*cat2 - k0*(inh2) + Cinh*bs1 + Cinh*bs3 + Cinh*bs4

f_cat2 = 2*k42*act2*(cF - cat2)/(cF - cat2 + cmin) - k9p*v2*cat2 - k10*cat2

f_v2 = 2*k12*act2*inh2 + k22*inh2 + k3*act2^2 - k9p*v2*cat2 - k1_3*v2 - k0*v2

f_act3 = -k13*act3*inh3 + k23*inh3 - 2*k3*act3^2 + k43*act3*(cF - cat3)/(cF - cat3 + cmin) - k0*act3

f_inh3 = -1*k13*act3*inh3 - k23*inh3 + k9p*v3*cat3 - k0*(inh3) + Cinh*bs2 + Cinh*bs1 + Cinh*bs4

f_cat3 = 2*k43*act3*(cF - cat3)/(cF - cat3 + cmin) - k9p*v3*cat3 - k10*cat3

f_v3 = 2*k13*act3*inh3 + k23*inh3 + k3*act3^2 - k9p*v3*cat3 - k1_3*v3 - k0*v3

f_act4 = -k14*act4*inh4 + k24*inh4 - 2*k3*act4^2 + k44*act4*(cF - cat4)/(cF - cat4 + cmin) - k0*act4

f_inh4 = -1*k14*act4*inh4 - k24*inh4 + k9p*v4*cat4 - k0*(inh4) + Cinh*bs3 + Cinh*bs1 + Cinh*bs2

f_cat4 = 2*k44*act4*(cF - cat4)/(cF - cat4 + cmin) - k9p*v4*cat4 - k10*cat4

f_v4 = 2*k14*act4*inh4 + k24*inh4 + k3*act4^2 - k9p*v4*cat4 - k1_3*v4 - k0*v4

f_actSig1 = -k15*actSig1*inhSig1 + k25*inhSig1 - 2*k3*actSig1^2 + k45*actSig1*(cF - catSig1)/(cF - catSig1 + cmin) - k0*actSig1

f_inhSig1 = -k15*actSig1*inhSig1 - k25*inhSig1 + k9p*vSig1*catSig1 - k0*(inhSig1-Inh003) - kdiff*inhSig1*AgSig1

f_catSig1 = 2*k45*actSig1*(cF - catSig1)/(cF - catSig1 + cmin) - k9p*vSig1*catSig1 - k10*catSig1

f_vSig1 = 2*k15*actSig1*inhSig1 + k25*inhSig1 + k3*actSig1^2 - k9p*vSig1*catSig1 - k1_3*vSig1 - k0*vSig1

f_AgSig1 = Cact*(bsSig11 + bsSig12) - kdiff*inhSig1*AgSig1

f_actSig2 = -k15*actSig2*inhSig2 + k25*inhSig2 - 2*k3*actSig2^2 + k45*actSig2*(cF - catSig2)/(cF - catSig2 + cmin) - k0*actSig2

f_inhSig2 = -k15*actSig2*inhSig2 - k25*inhSig2 + k9p*vSig2*catSig2 - k0*(inhSig2-Inh003) - kdiff*inhSig2*AgSig2

f_catSig2 = 2*k45*actSig2*(cF - catSig2)/(cF - catSig2 + cmin) - k9p*vSig2*catSig2 - k10*catSig2

f_vSig2 = 2*k15*actSig2*inhSig2 + k25*inhSig2 + k3*actSig2^2 - k9p*vSig2*catSig2 - k1_3*vSig2 - k0*vSig2

f_AgSig2 = Cact*(bsSig21 + bsSig23) - kdiff*inhSig2*AgSig2

f_actSig3 = -k15*actSig3*inhSig3 + k25*inhSig3 - 2*k3*actSig3^2 + k45*actSig3*(cF - catSig3)/(cF - catSig3 + cmin) - k0*actSig3

f_inhSig3 = -k15*actSig3*inhSig3 - k25*inhSig3 + k9p*vSig3*catSig3 - k0*(inhSig3-Inh003) - kdiff*inhSig3*AgSig3

f_catSig3 = 2*k45*actSig3*(cF - catSig3)/(cF - catSig3 + cmin) - k9p*vSig3*catSig3 - k10*catSig3

f_vSig3 = 2*k15*actSig3*inhSig3 + k25*inhSig3 + k3*actSig3^2 - k9p*vSig3*catSig3 - k1_3*vSig3 - k0*vSig3

f_AgSig3 = Cact*(bsSig31 + bsSig34) - kdiff*inhSig3*AgSig3

f_actSig4 = -k15*actSig4*inhSig4 + k25*inhSig4 - 2*k3*actSig4^2 + k45*actSig4*(cF - catSig4)/(cF - catSig4 + cmin) - k0*actSig4

f_inhSig4 = -k15*actSig4*inhSig4 - k25*inhSig4 + k9p*vSig4*catSig4 - k0*(inhSig4-Inh003) - kdiff*inhSig4*AgSig4

f_catSig4 = 2*k45*actSig4*(cF - catSig4)/(cF - catSig4 + cmin) - k9p*vSig4*catSig4 - k10*catSig4

f_vSig4 = 2*k15*actSig4*inhSig4 + k25*inhSig4 + k3*actSig4^2 - k9p*vSig4*catSig4 - k1_3*vSig4 - k0*vSig4

f_AgSig4 = Cact*(bsSig42 + bsSig43) - kdiff*inhSig4*AgSig4

f_actSig5 = -k15*actSig5*inhSig5 + k25*inhSig5 - 2*k3*actSig5^2 + k45*actSig5*(cF - catSig5)/(cF - catSig5 + cmin) - k0*actSig5

f_inhSig5 = -k15*actSig5*inhSig5 - k25*inhSig5 + k9p*vSig5*catSig5 - k0*(inhSig5-Inh003) - kdiff*inhSig5*AgSig5

f_catSig5 = 2*k45*actSig5*(cF - catSig5)/(cF - catSig5 + cmin) - k9p*vSig5*catSig5 - k10*catSig5

f_vSig5 = 2*k15*actSig5*inhSig5 + k25*inhSig5 + k3*actSig5^2 - k9p*vSig5*catSig5 - k1_3*vSig5 - k0*vSig5

f_AgSig5 = Cact*(bsSig52 + bsSig54) - kdiff*inhSig5*AgSig5

f_actSig6 = -k15*actSig6*inhSig6 + k25*inhSig6 - 2*k3*actSig6^2 + k45*actSig6*(cF - catSig6)/(cF - catSig6 + cmin) - k0*actSig6

f_inhSig6 = -k15*actSig6*inhSig6 - k25*inhSig6 + k9p*vSig6*catSig6 - k0*(inhSig6-Inh003) - kdiff*inhSig6*AgSig6

f_catSig6 = 2*k45*actSig6*(cF - catSig6)/(cF - catSig6 + cmin) - k9p*vSig6*catSig6 - k10*catSig6

f_vSig6 = 2*k15*actSig6*inhSig6 + k25*inhSig6 + k3*actSig6^2 - k9p*vSig6*catSig6 - k1_3*vSig6 - k0*vSig6

f_AgSig6 = Cact*(bsSig63 + bsSig64) - kdiff*inhSig6*AgSig6

{timers from reactors to reactors}

f_bss1 =b1*(-bss1^3 + (bsr1+bsr2)*bss1^2 - (bsr1*bsr2)*bss1) + 4*ustep(act1-{->}actspike{<-})*ustep(f_act1) - 0.6*ustep(pd1 - 0.5)

f_pd1 = 0.5*(bss1 - Qd1*pd1)/tau_d

f_bs1 = b2*(-bs1^3 + (bsr1+bsr2)*bs1^2 - (bsr1*bsr2)*bs1) + 1*ustep(pd1 - 0.5) - 0.8*ustep(pr1 - 0.6) - bs1*Qbs1

f_pr1 = 0.6*(bs1 - Qr1*pr1)/delta_t

f_bss2 =b1*(-bss2^3 + (bsr1+bsr2)*bss2^2 - (bsr1*bsr2)*bss2) + 4*ustep(act2-actspike)*ustep(f_act2) - 0.6*ustep(pd2 - 0.5)

f_pd2 = 0.5*(bss2 - Qd2*pd2)/tau_d

f_bs2 = b2*(-bs2^3 + (bsr1+bsr2)*bs2^2 - (bsr1*bsr2)*bs2) + 1*ustep(pd2 - 0.5) - 0.8*ustep(pr2 - 0.6) - bs2*Qbs2

f_pr2 = 0.6*(bs2 - Qr2*pr2)/delta_t

f_bss3 =b1*(-bss3^3 + (bsr1+bsr2)*bss3^2 - (bsr1*bsr2)*bss3) + 4*ustep(act3-actspike)*ustep(f_act3) - 0.6*ustep(pd3 - 0.5)

f_pd3 = 0.5*(bss3 - Qd3*pd3)/tau_d

f_bs3 = b2*(-bs3^3 + (bsr1+bsr2)*bs3^2 - (bsr1*bsr2)*bs3) + 1*ustep(pd3 - 0.5) - 0.8*ustep(pr3 - 0.6) - bs3*Qbs3

f_pr3 = 0.6*(bs3 - Qr3*pr3)/delta_t

f_bss4 =b1*(-bss4^3 + (bsr1+bsr2)*bss4^2 - (bsr1*bsr2)*bss4) + 4*ustep(act4-actspike)*ustep(f_act4) - 0.6*ustep(pd4 - 0.5)

f_pd4 = 0.5*(bss4 - Qd4*pd4)/tau_d

f_bs4 = b2*(-bs4^3 + (bsr1+bsr2)*bs4^2 - (bsr1*bsr2)*bs4) + 1*ustep(pd4 - 0.5) - 0.8*ustep(pr4 - 0.6) - bs4*Qbs4

f_pr4 = 0.6*(bs4 - Qr4*pr4)/delta_t

{1}

f_bssSig11 =b1*(-bssSig11^3 + (bsr1+bsr2)*bssSig11^2 - (bsr1*bsr2)*bssSig11) + 4*ustep(act1-actspike)*ustep(f_act1) - 0.6*ustep(pdSig11 - 0.5)

f_pdSig11 = 0.5*(bssSig11 - QdSig11*pdSig11)/(tau_d_coeff11 * period1)

f_bsSig11 = b2*(-bsSig11^3 + (bsr1+bsr2)*bsSig11^2 - (bsr1*bsr2)*bsSig11) + 1*ustep(pdSig11 - 0.5) - 0.8*ustep(prSig11 - 0.6) - bsSig11*QbsSig11

f_prSig11 = 0.6*(bsSig11 - QrSig11*prSig11)/delta_t

f_bssSig12 =b1*(-bssSig12^3 + (bsr1+bsr2)*bssSig12^2 - (bsr1*bsr2)*bssSig12) + 4*ustep(act2-actspike)*ustep(f_act2) - 0.6*ustep(pdSig12 - 0.5)

f_pdSig12 = 0.5*(bssSig12 - QdSig12*pdSig12)/(tau_d_coeff12 * period2)

f_bsSig12 = b2*(-bsSig12^3 + (bsr1+bsr2)*bsSig12^2 - (bsr1*bsr2)*bsSig12) + 1*ustep(pdSig12 - 0.5) - 0.8*ustep(prSig12 - 0.6) - bsSig12*QbsSig12

f_prSig12 = 0.6*(bsSig12 - QrSig12*prSig12)/delta_t

{2}

f_bssSig21 =b1*(-bssSig21^3 + (bsr1+bsr2)*bssSig21^2 - (bsr1*bsr2)*bssSig21) + 4*ustep(act1-actspike)*ustep(f_act1) - 0.6*ustep(pdSig21 - 0.5)

f_pdSig21 = 0.5*(bssSig21 - QdSig21*pdSig21)/(tau_d_coeff21 * period1)

f_bsSig21 = b2*(-bsSig21^3 + (bsr1+bsr2)*bsSig21^2 - (bsr1*bsr2)*bsSig21) + 1*ustep(pdSig21 - 0.5) - 0.8*ustep(prSig21 - 0.6) - bsSig21*QbsSig21

f_prSig21 = 0.6*(bsSig21 - QrSig21*prSig21)/delta_t

f_bssSig23 =b1*(-bssSig23^3 + (bsr1+bsr2)*bssSig23^2 - (bsr1*bsr2)*bssSig23) + 4*ustep(act3-actspike)*ustep(f_act3) - 0.6*ustep(pdSig23 - 0.5)

f_pdSig23 = 0.5*(bssSig23 - QdSig23*pdSig23)/(tau_d_coeff23 * period3)

f_bsSig23 = b2*(-bsSig23^3 + (bsr1+bsr2)*bsSig23^2 - (bsr1*bsr2)*bsSig23) + 1*ustep(pdSig23 - 0.5) - 0.8*ustep(prSig23 - 0.6) - bsSig23*QbsSig23

f_prSig23 = 0.6*(bsSig23 - QrSig23*prSig23)/delta_t

{3}

f_bssSig31 =b1*(-bssSig31^3 + (bsr1+bsr2)*bssSig31^2 - (bsr1*bsr2)*bssSig31) + 4*ustep(act1-actspike)*ustep(f_act1) - 0.6*ustep(pdSig31 - 0.5)

f_pdSig31 = 0.5*(bssSig31 - QdSig31*pdSig31)/(tau_d_coeff31 * period1)

f_bsSig31 = b2*(-bsSig31^3 + (bsr1+bsr2)*bsSig31^2 - (bsr1*bsr2)*bsSig31) + 1*ustep(pdSig31 - 0.5) - 0.8*ustep(prSig31 - 0.6) - bsSig31*QbsSig31

f_prSig31 = 0.6*(bsSig31 - QrSig31*prSig31)/delta_t

f_bssSig34 =b1*(-bssSig34^3 + (bsr1+bsr2)*bssSig34^2 - (bsr1*bsr2)*bssSig34) + 4*ustep(act4-actspike)*ustep(f_act4) - 0.6*ustep(pdSig34 - 0.5)

f_pdSig34 = 0.5*(bssSig34 - QdSig34*pdSig34)/(tau_d_coeff34 * period4)

f_bsSig34 = b2*(-bsSig34^3 + (bsr1+bsr2)*bsSig34^2 - (bsr1*bsr2)*bsSig34) + 1*ustep(pdSig34 - 0.5) - 0.8*ustep(prSig34 - 0.6) - bsSig34*QbsSig34

f_prSig34 = 0.6*(bsSig34 - QrSig34*prSig34)/delta_t

{4}

f_bssSig42 =b1*(-bssSig42^3 + (bsr1+bsr2)*bssSig42^2 - (bsr1*bsr2)*bssSig42) + 4*ustep(act2-actspike)*ustep(f_act2) - 0.6*ustep(pdSig42 - 0.5)

f_pdSig42 = 0.5*(bssSig42 - QdSig42*pdSig42)/(tau_d_coeff42 * period2)

f_bsSig42 = b2*(-bsSig42^3 + (bsr1+bsr2)*bsSig42^2 - (bsr1*bsr2)*bsSig42) + 1*ustep(pdSig42 - 0.5) - 0.8*ustep(prSig42 - 0.6) - bsSig42*QbsSig42

f_prSig42 = 0.6*(bsSig42 - QrSig42*prSig42)/delta_t

f_bssSig43 =b1*(-bssSig43^3 + (bsr1+bsr2)*bssSig43^2 - (bsr1*bsr2)*bssSig43) + 4*ustep(act3-actspike)*ustep(f_act3) - 0.6*ustep(pdSig43 - 0.5)

f_pdSig43 = 0.5*(bssSig43 - QdSig43*pdSig43)/(tau_d_coeff43 * period3)

f_bsSig43 = b2*(-bsSig43^3 + (bsr1+bsr2)*bsSig43^2 - (bsr1*bsr2)*bsSig43) + 1*ustep(pdSig43 - 0.5) - 0.8*ustep(prSig43 - 0.6) - bsSig43*QbsSig43

f_prSig43 = 0.6*(bsSig43 - QrSig43*prSig43)/delta_t

{5}

f_bssSig52 =b1*(-bssSig52^3 + (bsr1+bsr2)*bssSig52^2 - (bsr1*bsr2)*bssSig52) + 4*ustep(act2-actspike)*ustep(f_act2) - 0.6*ustep(pdSig52 - 0.5)

f_pdSig52 = 0.5*(bssSig52 - QdSig52*pdSig52)/(tau_d_coeff52 * period2)

f_bsSig52 = b2*(-bsSig52^3 + (bsr1+bsr2)*bsSig52^2 - (bsr1*bsr2)*bsSig52) + 1*ustep(pdSig52 - 0.5) - 0.8*ustep(prSig52 - 0.6) - bsSig52*QbsSig52

f_prSig52 = 0.6*(bsSig52 - QrSig52*prSig52)/delta_t

f_bssSig54 =b1*(-bssSig54^3 + (bsr1+bsr2)*bssSig54^2 - (bsr1*bsr2)*bssSig54) + 4*ustep(act4-actspike)*ustep(f_act4) - 0.6*ustep(pdSig54 - 0.5)

f_pdSig54 = 0.5*(bssSig54 - QdSig54*pdSig54)/(tau_d_coeff54 * period3)

f_bsSig54 = b2*(-bsSig54^3 + (bsr1+bsr2)*bsSig54^2 - (bsr1*bsr2)*bsSig54) + 1*ustep(pdSig54 - 0.5) - 0.8*ustep(prSig54 - 0.6) - bsSig54*QbsSig54

f_prSig54 = 0.6*(bsSig54 - QrSig54*prSig54)/delta_t

{6}

f_bssSig63 =b1*(-bssSig63^3 + (bsr1+bsr2)*bssSig63^2 - (bsr1*bsr2)*bssSig63) + 4*ustep(act3-actspike)*ustep(f_act3) - 0.6*ustep(pdSig63 - 0.5)

f_pdSig63 = 0.5*(bssSig63 - QdSig63*pdSig63)/(tau_d_coeff63 * period2)

f_bsSig63 = b2*(-bsSig63^3 + (bsr1+bsr2)*bsSig63^2 - (bsr1*bsr2)*bsSig63) + 1*ustep(pdSig63 - 0.5) - 0.8*ustep(prSig63 - 0.6) - bsSig63*QbsSig63

f_prSig63 = 0.6*(bsSig63 - QrSig63*prSig63)/delta_t

f_bssSig64 =b1*(-bssSig64^3 + (bsr1+bsr2)*bssSig64^2 - (bsr1*bsr2)*bssSig64) + 4*ustep(act4-actspike)*ustep(f_act4) - 0.6*ustep(pdSig64 - 0.5)

f_pdSig64 = 0.5*(bssSig64 - QdSig64*pdSig64)/(tau_d_coeff64 * period3)

f_bsSig64 = b2*(-bsSig64^3 + (bsr1+bsr2)*bsSig64^2 - (bsr1*bsr2)*bsSig64) + 1*ustep(pdSig64 - 0.5) - 0.8*ustep(prSig64 - 0.6) - bsSig64*QbsSig64

f_prSig64 = 0.6*(bsSig64 - QrSig64*prSig64)/delta_t

INITIAL VALUE

Act1 = Act10

Inh1 = Inh10

Cat1 = Cat10

v1 = v10

Act2 = Act20

Inh2 = Inh20

Cat2 = Cat20

v2 = v20

Act3 = Act30

Inh3 = Inh30

Cat3 = Cat30

v3 = v30

Act4 = Act40

Inh4 = Inh40

Cat4 = Cat40

v4 = v40

ActSig1 = ActSig1_0

InhSig1 = InhSig1_0

CatSig1 = CatSig1_0

vSig1 = vSig1_0

AgSig1 = AgSig1_0

ActSig2 = ActSig1_0

InhSig2 = InhSig1_0

CatSig2 = CatSig1_0

vSig2 = vSig1_0

AgSig2 = AgSig1_0

ActSig3 = ActSig1_0

InhSig3 = InhSig1_0

CatSig3 = CatSig1_0

vSig3 = vSig1_0

AgSig3 = AgSig1_0

ActSig4 = ActSig1_0

InhSig4 = InhSig1_0

CatSig4 = CatSig1_0

vSig4 = vSig1_0

AgSig4 = AgSig1_0

ActSig5 = ActSig1_0

InhSig5 = InhSig1_0

CatSig5 = CatSig1_0

vSig5 = vSig1_0

AgSig5 = AgSig1_0

ActSig6 = ActSig1_0

InhSig6 = InhSig1_0

CatSig6 = CatSig1_0

vSig6 = vSig1_0

AgSig6 = AgSig1_0

bs1 = 0

pr1= 0

bs2 = 0

pr2= 0

bs3 = 0

pr3= 0

bs4 = 0

pr4= 0

bss1=0

pd1=0

bss2=0

pd2=0

bss3=0

pd3=0

bss4=0

pd4=0

bsSig11 = 0

prSig11= 0

bsSig12 = 0

prSig12= 0

bsSig21 = 0

prSig21= 0

bsSig23 = 0

prSig23= 0

bsSig31 = 0

prSig31= 0

bsSig34 = 0

prSig34= 0

bsSig42 = 0

prSig42= 0

bsSig43 = 0

prSig43= 0

bsSig52 = 0

prSig52= 0

bsSig54 = 0

prSig54= 0

bsSig63 = 0

prSig63= 0

bsSig64 = 0

prSig64= 0

bssSig11=0

pdSig11=0

bssSig12=0

pdSig12=0

bssSig21=0

pdSig21=0

bssSig23=0

pdSig23=0

bssSig31=0

pdSig31=0

bssSig34=0

pdSig34=0

bssSig42=0

pdSig42=0

bssSig43=0

pdSig43=0

bssSig52=0

pdSig52=0

bssSig54=0

pdSig54=0

bssSig63=0

pdSig63=0

bssSig64=0

pdSig64=0

EQUATIONS

Act1: dt(Act1) = f_act1

Inh1: dt(Inh1) = f_inh1

cat1: dt(cat1) = f_cat1

v1: dt(v1) = f_v1

Act2: dt(Act2) = f_act2

Inh2: dt(Inh2) = f_inh2

cat2: dt(cat2) = f_cat2

v2: dt(v2) = f_v2

Act3: dt(Act3) = f_act3

Inh3: dt(Inh3) = f_inh3

cat3: dt(cat3) = f_cat3

v3: dt(v3) = f_v3

Act4: dt(Act4) = f_act4

Inh4: dt(Inh4) = f_inh4

cat4: dt(cat4) = f_cat4

v4: dt(v4) = f_v4

ActSig1: dt(ActSig1) = f_actSig1

InhSig1: dt(InhSig1) = f_inhSig1

catSig1: dt(catSig1) = f_catSig1

vSig1: dt(vSig1) = f_vSig1

agSig1: dt(agSig1) = f_agSig1

ActSig2: dt(ActSig2) = f_actSig2

InhSig2: dt(InhSig2) = f_inhSig2

catSig2: dt(catSig2) = f_catSig2

vSig2: dt(vSig2) = f_vSig2

agSig2: dt(agSig2) = f_agSig2

ActSig3: dt(ActSig3) = f_actSig3

InhSig3: dt(InhSig3) = f_inhSig3

catSig3: dt(catSig3) = f_catSig3

vSig3: dt(vSig3) = f_vSig3

agSig3: dt(agSig3) = f_agSig3

ActSig4: dt(ActSig4) = f_actSig4

InhSig4: dt(InhSig4) = f_inhSig4

catSig4: dt(catSig4) = f_catSig4

vSig4: dt(vSig4) = f_vSig4

agSig4: dt(agSig4) = f_agSig4

ActSig5: dt(ActSig5) = f_actSig5

InhSig5: dt(InhSig5) = f_inhSig5

catSig5: dt(catSig5) = f_catSig5

vSig5: dt(vSig5) = f_vSig5

agSig5: dt(agSig5) = f_agSig5

ActSig6: dt(ActSig6) = f_actSig6

InhSig6: dt(InhSig6) = f_inhSig6

catSig6: dt(catSig6) = f_catSig6

vSig6: dt(vSig6) = f_vSig6

agSig6: dt(agSig6) = f_agSig6

BS1: dt(bs1) = f_bs1

PR1: dt(pr1) = f_pr1

BS2: dt(bs2) = f_bs2

PR2: dt(pr2) = f_pr2

BS3: dt(bs3) = f_bs3

PR3: dt(pr3) = f_pr3

BS4: dt(bs4) = f_bs4

PR4: dt(pr4) = f_pr4

BSSIG11: dt(bsSig11) = f_bsSig11

PRSIG11: dt(prSig11) = f_prSig11

BSSIG12: dt(bsSig12) = f_bsSig12

PRSIG12: dt(prSig12) = f_prSig12

BSSIG21: dt(bsSig21) = f_bsSig21

PRSIG21: dt(prSig21) = f_prSig21

BSSIG23: dt(bsSig23) = f_bsSig23

PRSIG23: dt(prSig23) = f_prSig23

BSSIG31: dt(bsSig31) = f_bsSig31

PRSIG31: dt(prSig31) = f_prSig31

BSSIG34: dt(bsSig34) = f_bsSig34

PRSIG34: dt(prSig34) = f_prSig34

BSSIG42: dt(bsSig42) = f_bsSig42

PRSIG42: dt(prSig42) = f_prSig42

BSSIG43: dt(bsSig43) = f_bsSig43

PRSIG43: dt(prSig43) = f_prSig43

BSSIG52: dt(bsSig52) = f_bsSig52

PRSIG52: dt(prSig52) = f_prSig52

BSSIG54: dt(bsSig54) = f_bsSig54

PRSIG54: dt(prSig54) = f_prSig54

BSSIG63: dt(bsSig63) = f_bsSig63

PRSIG63: dt(prSig63) = f_prSig63

BSSIG64: dt(bsSig64) = f_bsSig64

PRSIG64: dt(prSig64) = f_prSig64

BSS1: dt(bss1) = f_bss1

PD1: dt(pd1) = f_pd1

BSS2: dt(bss2) = f_bss2

PD2: dt(pd2) = f_pd2

BSS3: dt(bss3) = f_bss3

PD3: dt(pd3) = f_pd3

BSS4: dt(bss4) = f_bss4

PD4: dt(pd4) = f_pd4

BSSSIG11: dt(bssSig11) = f_bssSig11

PDSIG11: dt(pdSig11) = f_pdSig11

BSSSIG12: dt(bssSig12) = f_bssSig12

PDSIG12: dt(pdSig12) = f_pdSig12

BSSSIG21: dt(bssSig21) = f_bssSig21

PDSIG21: dt(pdSig21) = f_pdSig21

BSSSIG23: dt(bssSig23) = f_bssSig23

PDSIG23: dt(pdSig23) = f_pdSig23

BSSSIG31: dt(bssSig31) = f_bssSig31

PDSIG31: dt(pdSig31) = f_pdSig31

BSSSIG34: dt(bssSig34) = f_bssSig34

PDSIG34: dt(pdSig34) = f_pdSig34

BSSSIG42: dt(bssSig42) = f_bssSig42

PDSIG42: dt(pdSig42) = f_pdSig42

BSSSIG43: dt(bssSig43) = f_bssSig43

PDSIG43: dt(pdSig43) = f_pdSig43

BSSSIG52: dt(bssSig52) = f_bssSig52

PDSIG52: dt(pdSig52) = f_pdSig52

BSSSIG54: dt(bssSig54) = f_bssSig54

PDSIG54: dt(pdSig54) = f_pdSig54

BSSSIG63: dt(bssSig63) = f_bssSig63

PDSIG63: dt(pdSig63) = f_pdSig63

BSSSIG64: dt(bssSig64) = f_bssSig64

PDSIG64: dt(pdSig64) = f_pdSig64

BOUNDARIES

region 1

start (0) line to (1)

time time_ini to time_end by 0.00000001

MONITORS

for t = 0 by 100 to time_end

PLOTS

for t = 0 by 500 to time_end

history(act1, act2, act3, act4) at (0.5,0.5) export format "#t#r#b#i" file="result\"+oscs_path+"1 act.txt"

history(actSig1) at (0.5,0.5) export format "#t#r#b#i" file="result\"+oscs_path+"1 12 signal set to\5 actSignal.txt"

history(actSig2) at (0.5,0.5) export format "#t#r#b#i" file="result\"+oscs_path+"2 13 signal set to\5 actSignal.txt"

history(actSig3) at (0.5,0.5) export format "#t#r#b#i" file="result\"+oscs_path+"3 14 signal set to\5 actSignal.txt"

history(actSig4) at (0.5,0.5) export format "#t#r#b#i" file="result\"+oscs_path+"4 23 signal set to\5 actSignal.txt"

history(actSig5) at (0.5,0.5) export format "#t#r#b#i" file="result\"+oscs_path+"5 24 signal set to\5 actSignal.txt"

history(actSig6) at (0.5,0.5) export format "#t#r#b#i" file="result\"+oscs_path+"6 34 signal set to\5 actSignal.txt"

END
